# Autoencoders for genomic variation analysis

**DOI:** 10.1101/gr.280086.124

**Published:** 2026-02

**Authors:** Margarita Geleta, Daniel Mas Montserrat, Xavier Giro-i-Nieto, Alexander G. Ioannidis

**Affiliations:** 1 Department of Biomedical Data Science, Stanford University School of Medicine, Stanford, California 94305, USA;; 2 Department of Signal and Theory Communications, Universitat Politècnica de Catalunya, Barcelona 08034, Spain;; 3 Department of Electrical Engineering and Computer Science, University of California, Berkeley, Berkeley, California 94720, USA;; 4 Genomics Institute, University of California, Santa Cruz, Santa Cruz, California 95060, USA;; 5 Institute for Computational and Mathematical Engineering, Stanford University School of Engineering, Stanford, California 94305, USA

## Abstract

Modern biobanks are providing numerous high-resolution genomic sequences of diverse populations. In order to account for diverse and admixed populations, new algorithmic tools are needed in order to properly capture the genetic composition of populations. Here, we explore deep learning techniques, namely, variational autoencoders (VAEs), to process genomic data from a population perspective. We show the power of VAEs for a variety of tasks relating to the interpretation, compression, classification, and simulation of genomic data with several worldwide whole genome data sets from both humans and canids, and evaluate the performance of the proposed applications with and without ancestry conditioning. The unsupervised setting of autoencoders allows for the detection and learning of granular population structure and inferring of informative latent factors. The learned latent spaces of VAEs are able to capture and represent differentiated Gaussian-like clusters of samples with similar genetic composition on a fine scale from single nucleotide polymorphisms (SNPs), enabling applications in dimensionality reduction and data simulation. These individual genotype sequences can then be decomposed into latent representations and reconstruction errors (residuals), which provide a sparse representation useful for lossless compression. We show that different populations have differentiated compression ratios and classification accuracies. Additionally, we analyze the entropy of the SNP data, its effect on compression across populations, and its relation to historical migrations, and we show how to introduce autoencoders into existing compression pipelines.

## Introduction

Deep learning is becoming ubiquitous across all areas of science and engineering, with artificial neural networks (ANNs) being used to model highly nonlinear and complex data and proving successful in a wide range of applications. Recent works have begun to introduce such techniques within the fields of population genetics and precision medicine (Romero et al. 2016; Montserrat et al. 2020; Dominguez Mantes et al. 2023). Here, we explore the use of variational autoencoders (VAEs), a type of neural network used to learn a low-dimensional representation of the data, to analyze sequences of single nucleotide polymorphisms (SNPs) and showcase many applications including dimensionality reduction, data compression, classification, and simulation.

The number of human genomes being sequenced every year is growing rapidly, fueled by improvements in sequencing technology (Hernaez et al. 2019). Biobanks, paramount in areas like precision medicine, are powering genome-wide association studies (GWAS), where many genetic variants (e.g., SNPs) are analyzed across different subjects to find the relationships between genetic and phenotypic traits (Mardis 2011; Wojcik et al. 2019), are used to develop new treatments and drugs (Shah and Gaedigk 2018), and to address disparities and promote equity in precision medicine (Rhead et al. 2023). This creates a need for new, efficient, and accurate data-driven algorithmic tools to store, visualize, and characterize high-dimensional genomic data. Whereas many traditional statistical techniques for genomic data like hidden Markov models (HMMs) (Tang et al. 2006; Corbett-Detig and Nielsen 2017; Browning et al. 2018, 2023) become computationally expensive and slow when faced with biobank-sized data, neural networks can provide a powerful alternative.

### Genomic sequence compression

The storage and transmission of high-dimensional biobank data require substantial space and channel capacity. This need has motivated the development of high-performance compression tools tailored to the unique particularities of this type of data (Brandon et al. 2009; Giancarlo et al. 2009; Nalbantoglu et al. 2010; Hernaez et al. 2019), which include the inherently high dimensionality of the data and its sparse yet complex structure. The demand for more powerful compression approaches becomes increasingly vital in modern large biobanks that contain genomic sequences produced by high-throughput sequencing. ANN-based approaches had already shown superior compression ratios decades ago (Schmidhuber and Heil 1996; Mahoney 2000). The first neural compressors mimicked the adaptive prediction by partial matching (PPM) model, where a neural network is used to estimate (and/or adjust) the probabilities for each symbol and then a usual coding method such as arithmetic coding is used to convert the data into a compressed bitstream (Mahoney 2000). Following this line, “DeepZip” (Goyal et al. 2018) estimated the probabilities by processing genomic sequences with gated recurrent units (GRUs) and long short-term memory units (LSTMs). Mahoney (2005) introduced “logistic mixing,” a neural network without hidden layers that uses a simple update rule to adjust the output probabilities for better bitstream coding. This idea has been brought to genomic compression of sequencing reads with GeCo3 (Silva et al. 2020). Another recent use of neural networks for DNA compression has been shown in DeepDNA (Wang et al. 2018), trained specifically on mitochondrial DNA data, using a convolutional layer to capture local features which are then combined and fed into a recurrent layer to output the probabilities for each symbol. On the other hand, autoencoders present an alternative to PPM. The first attempt to compress genomic data with simple autoencoders has been performed on sequencing reads data (Absardi and Javidan 2019), followed by a more sophisticated autoencoder-based method, GenCoder (Sheena and Nair 2024). To the authors’ knowledge, there is no documented research leveraging autoencoders to losslessly compress SNP data sets.

### Ancestry prediction

The relationship between ancestry, ethnicity, and genetic variation is complex, involving genetics, history, and society. Genetic variation does not follow identities established culturally and historically in a simple way (Roberts 2011). The term *ancestry* refers to a class of genetic similarity that can be associated with a shared origin (Xie et al. 2001; Fujimura and Rajagopalan 2011). The framework for classifying or regressing genetic ancestry uses the genome sequence for its features (Nelson et al. 2018). Indeed, an individual’s ancestral geographic origin can be inferred with remarkable accuracy from their DNA (Novembre et al. 2008). This task, known as *global ancestry inference*, can be either approached from a discrete perspective (classifying the ancestry label) or from a continuous one (regressing the geographical coordinates of origin). Some widely adopted techniques include ADMIXTURE (Pritchard et al. 2000; Alexander et al. 2009), a clustering technique based on probabilistic non-negative matrix factorization, and its more recent neural counterpart—Neural ADMIXTURE (Dominguez Mantes et al. 2023); Locator (Battey et al. 2020), a multilayer perceptron (MLP) that addresses the *geographical coordinate regression* problem by estimating a nonlinear function mapping genotypes to locations; and Diet Networks (Romero et al. 2016), which represent another deep-learning-based ancestry classifier within this paradigm of methods. Nevertheless, characterizing ancestry as a set of discrete, predefined labels can be limiting, as individuals are at some level all admixed, stemming from ancestors belonging to multiple ancestral population groups (Supplemental Methods S1). For instance, the genomes of numerous African-Americans have variable proportions of segments that could be classified as European and West African ancestry (Ali-Khan and Daar 2010). To characterize these different genomic segments, one can use local ancestry inference (LAI) methods like RFMix (Maples et al. 2013). These can rely on neural networks as well, for instance LAI-Net (Montserrat et al. 2020) and SALAI-Net (Sabat Oriol et al. 2022).

### Genomic sequence simulation

Although the number of sequenced genomes has grown substantially over the years, there is a clear disparity among the ancestries represented. The proportion of participants of non-European descent has remained constant (Wojcik et al. 2019), potentially introducing bias toward European genomes and giving rise to the “missing diversity” problem (Popejoy and Fullerton 2016). To illustrate, as of 2018, the majority of GWAS encompassed approximately 78% individuals of European ancestry. Additionally, certain communities, predominantly composed of individuals with non-European ancestry, are reluctant to participate in genetic studies due to privacy concerns or apprehensions about potential misuse, as seen in prior cases (Maher 2015; Guglielmi 2019). To circumvent these challenges, data simulation tools can be used to augment genomic databases and offer mechanisms for sharing synthetic data possessing equivalent statistical properties, all while safeguarding the privacy of individuals. Several recent studies have explored the effectiveness of generative deep neural networks in generating simulated genotypes. Montserrat et al. (2019) use a class-conditional VAE-GAN to generate artificial yet realistic genotypes, whereas Yelmen et al. (2021) generate high-quality synthetic genomes with GAN and RBM. Moment matching networks have provided competitive results for data simulation (Perera et al. 2022). Furthermore, another work by Battey et al. (2021) has attempted to use VAEs for genotype simulation.

To summarize the contributions: In this work, we demonstrate how a single model can address several critical tasks in genomics research. By leveraging the nonlinearities and the modular architecture of our VAE, we implicitly model linkage disequilibrium (LD) and facilitate interpretable latent structures. First, we illustrate how a VAE can be employed for lossless compression by storing the latent representation of the SNP sequences and their corresponding compressed residuals for error correction at decoding time. Our method represents the first instance of integrating autoencoders in existing compression pipelines, increasing the compression factors of large SNP data sets. Second, we introduce an ancestry-conditioned formulation of the VAEs and provide both qualitative and quantitative evaluations of the clustering quality in the latent space, comparing it to the commonly used principal component analysis (PCA), which still remains a strong baseline in population genetics (Tan and Atkinson 2023). Finally, we present an elegant method for sampling new SNP sequences from the modeled distribution, conditioned on ancestry, and contrast several metrics (including the LD structure and the SNP entropy) of the simulated sequences with that of real sequences as a genotype simulation quality assessment.

## Methods

### Variational autoencoders

Representation learning, also known as *feature learning*, attempts to recover a compact set of so-called latent *q*-dimensional variables ***z*** that describe a distribution over the *d*-dimensional observed data ***x***, with *q* < *d*. PCA is a well-established statistical procedure for dimensionality reduction and widely used in the population genetics community (Tan and Atkinson 2023). For a set of observed data ***x***, the latent variables ***z*** are the orthonormal axes onto which the retained variance under projection of the data points is maximal. PCA can be given a natural probabilistic interpretation as the dimensionality reduction process can be considered in terms of the distribution of the latent variables, conditioned on the observation (Tipping and Bishop 1999), where, from factor analysis, the relationship between ***x*** and ***z*** is linear and, conventionally, Gaussianity assumptions are taken. However, there are cases where the relationship between ***x*** and ***z*** is not linear and the common simplifying assumptions about Gaussianity of the marginal or posterior probabilities do not reflect real data. In those cases, autoencoders are a perfect fit because they learn a direct encoding—a parametric map from inputs ***x*** to their latent representation ***z***, becoming a nonlinear generalization of PCA (Hinton and Salakhutdinov 2006). In this setup, two closed-form parametrized functions are defined: (a) the *encoder* $V_{e}\;:\;X\to Z$ and (b) the *decoder* $V_{d}\;:\;Z\to X$. Both $V_{e}(\cdot )$ and $V_{d}(\cdot )$ can be as expressive as desired: from a single linear layer to a MLP, or any other ANN architecture. In VAEs for SNP modeling, the input $x\in B^{d}$ is encoded with $V_{e}(\cdot )$ into the mean ***μ*** and a function of the variance ***σ*** vectors. Reparametrizing, the latent representation z=μ+σ⊙ϵ is obtained, with ϵ∼N(0,I), ⊙ being the elementwise product, and $z,\mu ,\sigma ,\epsilon \in R^{q}$. The latent representation can then be decoded with the decoder $V_{d}(\cdot )$ to obtain the reconstruction $\hat{x}=1_{1/2}(o)$, $\hat{x}\in B^{d}$, where $o=V_{d}(V_{e}(x))\in {\left[0,1\right]}^{d}$ is the output of the network and $1_{1/2}(\cdot )$ is a unit step function applied elementwise on the output, binarizing each element of the output with a threshold of 1/2. We define the composition of the encoder–decoder pair and the binarization layer as $f_{\theta }=1_{1/2}\circ V_{d}\circ V_{e}$, with parameters ***θ***, such that $\hat{x}=f_{\theta }(x)$, and because our input is always a sequence of binary values, we have $f_{\theta }\;:\;B^{d}\to B^{d}$. We adopt an under-complete architecture, in which the latent representation between the encoder and the decoder (traditionally referred to as the *bottleneck*; Vincent et al. 2010) has a smaller dimensionality than the input (*q* < *d*). In this setup, the primary learning objective is to compel the encoder to preserve as much of the relevant information as possible within this limited dimensionality.

Autoencoders learn the mapping function from input to feature space (the space spanned by ***z***) and the reverse mapping without learning an explicit probability distribution of the data. In contrast, VAEs learn a probability distribution of the data (Kingma and Welling 2014) by enforcing an isotropic Gaussian prior over the latent variables. Because they learn to model the data, new samples can be generated by sampling, meaning that VAE are *generative* autoencoders (Supplemental Methods S2, S5, and S6).

### Ancestry-conditional VAEs

Note that the above approach does not condition VAE on ancestry labels during training—it trains on all populations together. However, an ancestry-conditioned VAE includes the additional information of the population label *y*_*n*_ for each input sample ***x***_*n*_. This means we need to account for the labeled data set $D=(D_{x},D_{y})$ in the loss function, where data set $D_{x}=\{x_{n}\;|\;1\leq n\leq N\}$ is the set of *d*-dimensional samples and $D_{y}=\{y_{n}\;|\;1\leq n\leq N,y_{n}\in Y\}$ is the set of corresponding ancestry labels, where Y is the set of populations present in the data. After incorporating ancestry conditioning in Equation 17, and following a similar derivation as in Equations 18 and 19 (Supplemental Methods S6), we arrive at the same generative loss objective for the ancestry-conditioned VAE:

(1) $$\begin{array}{rl} \;p(D_{x}\;|\;D_{y},\theta ) & =\prod_{n=1}^{N}p(x_{n}\;|\;y_{n},\theta )\\ & =\prod_{k\in Y}\prod_{n\;:\;y_{n}=k}p(x_{n}\;|\;Y=k,\theta ). \end{array}$$

This indicates that the objective for the generative loss remains consistent in the ancestry-conditioned VAE:

(2) $$\log p(D_{x}\;|\;D_{y},\theta )=-\sum_{k\in Y}\sum_{n\;:\;y_{n}=k}\ell_{\mathrm{BCE}}(x_{n},o_{n}^{\left(k\right)}),$$

denoting the VAE output conditioned on ancestry label *k* as $o_{n}^{\left(k\right)}=f_{\theta }^{\left(k\right)}(x_{n})$. In practical terms, there are different ways to incorporate conditioning on the *k*th ancestry into both the encoder and the decoder, which we denote as $V_{e}^{\left(k\right)}(\cdot )$ and $V_{d}^{\left(k\right)}(\cdot )$, respectively. One approach, which we refer to as *regular* C-VAE (conditioned VAE), appends a one-hot encoded ancestry label to both the encoder and the decoder. An alternative approach for conditioning fits a separate VAE for each population group individually, resulting in ancestry-specific overfitted VAEs. We refer to this approach as Y-VAE (Y-overfitted VAE), a method which has |Y| number of times more parameters than a regular VAE.

### Bayesian-motivated classification objectives

When employing ancestry conditioning on a VAE, an ancestry label can be inferred through maximum a posteriori (MAP) estimation. The output of the VAE conditioned on ancestry *k* is a vector containing Bernoulli probabilities $o_{n}^{\left(k\right)}\in R^{d}$. To infer an ancestry label, this vector is thresholded with a value greater than 1/2, resulting in ${\hat{x}}_{n}$. Because each SNP position is independently modeled as a Bernoulli distribution, the joint distribution can be expressed as the product of individual SNP distributions. Applying Bayes’ rule, we can derive *p*(*Y* = *k* | ***x***_*n*_) ∝ *p*(***x***_*n*_ | *Y* = *k*)*p*(*Y* = *k*), where *p*(***x***_*n*_ | *Y* = *k*) represents the Bernoulli likelihood and the prior *p*(*Y* = *k*), for simplicity, is defined as the categorical distribution over *K* ancestry labels because the data used for the classification task have uniformly distributed ancestry labels. Therefore,

(3) $$p(Y=k|x_{n})\propto \prod_{i=1}^{d}{\left(o_{ni}^{\left(k\right)}\right)}^{x_{ni}}{\left(1-o_{ni}^{\left(k\right)}\right)}^{\left(1-x_{ni}\right)}.$$

Given that *d* represents a relatively large number of dimensions, numerical computation can lead to the entire expression collapsing to zero. This effect is due to two factors. First, if at least one SNP position is reconstructed incorrectly, that is, the true value of the SNP position is 0 but the VAE returns 1 or vice versa, the expression becomes zero. Second, when many positions have extremely small values due to numerical precision limitations, the product of these values becomes effectively zero. To address these issues and improve numerical stability, it is common practice to operate in the space of log-probabilities. We therefore apply logarithms to Equation 3:

(4) $$\begin{array}{rl} \log\;p(Y=k\;|\;x_{n}) & \;\propto \sum_{i=1}^{d}x_{ni}\log\left(o_{ni}^{\left(k\right)}\right)+(1-x_{ni})\log\left(1-o_{ni}^{\left(k\right)}\right)\\ & \;=-\ell_{\mathrm{BCE}}(x_{n},o_{n}^{\left(k\right)}). \end{array}$$

Selecting the ancestry label that maximizes the posterior *p*(*Y* = *k* | ***x***_*n*_) is equivalent to minimizing the BCE loss:

(5) $$y_{n}=\underset{k\in Y}{\arg\;\max}p(Y=k|x_{n})=\underset{k\in Y}{\arg\;\min}\ell_{\mathrm{BCE}}(x_{n},o_{n}^{\left(k\right)}).$$

To address the issues associated with the logarithm of zero and the potential misclassification of SNP positions, some form of smoothing or clamping is necessary. One way to implement a form of *Laplace smoothing* is by adjusting the sigmoid temperature, which makes the Bernoulli probabilities $o_{ni}^{\left(k\right)}$ less sharp and less likely to be clamped to zero quickly. However, even with this approach, there can still be issues with zeroing. To mitigate this, we employ the trick used in the PyTorch implementation of the BCE loss (Paszke et al. 2019). This involves clamping the probabilities to a certain value if they go beyond a specified threshold. Specifically, we clamp *p*(*Y* = *k* | ***x***_*n*_) to *e*^−100^. In terms of logarithms, this is equivalent to clamping $\log p(Y=k\;|\;x_{n})$ to −100. This approach ensures that the loss remains finite and avoids the issues associated with infinite values due to the logarithm of zero.

Another important conclusion is that the Bernoulli likelihood maximization problem can be reduced to the minimization of the *L*_1_ discrepancy between the input and the output of the VAE: Let ℓ_1_(***x***, ***o***^(*k*)^) = ||***x*** − ***o***^(*k*)^||_1_ be the *L*_1_ discrepancy between the SNP array ***x*** and its Bernoulli probabilities by VAE conditioned on *k*th ancestry. And let *b*(***x***, ***o***^(*k*)^) be the multivariate i.i.d. Bernoulli likelihood function. Then, for each *i*th element in ***x***:

- $\ell_{1}(x_{i},o_{i}^{\left(k\right)})=o_{i}^{\left(k\right)}$ and $b_{i}(x_{i},o_{i}^{\left(k\right)})=1-o_{i}^{\left(k\right)}$ if *x*_*i*_ = 0.
- $\ell_{1}(x_{i},o_{i}^{\left(k\right)})=1-o_{i}^{\left(k\right)}$ and $b_{i}(x_{i},o_{i}^{\left(k\right)})=o_{i}^{\left(k\right)}$ if *x*_*i*_ = 1.

Thus, $b_{i}(x_{i},o_{i}^{\left(k\right)})=1-\ell_{1}(x_{i},o_{i}^{\left(k\right)})$, from which follows the expression

(6) $$b(x,o^{\left(k\right)})=\underset{i}{\prod }\left(1-\ell_{1}(x_{i},o_{i}^{\left(k\right)})\right).$$

Taking logarithm on Equation 6 yields $\log b(x,o^{\left(k\right)})\;=$ $\sum_{i}\log\left(1-\ell_{1}(x_{i},o_{i}^{\left(k\right)})\right)$. The Taylor series of log⁡(1−z) about zero is

(7) $$\begin{array}{rl} \log\left(1-z\right) & \;\approx \log1+\frac{\partial }{\partial z}\log\left(1-z\right)z+O(z^{2})\\ & \;=-z+O(z^{2}). \end{array}$$

For small *z*, all terms of order *z*^2^ are negligible and we can employ the approximation log⁡(1−z)≈−z. Therefore, arg min ℓ_BCE_(***x***, ***o***^(*k*)^) can be approximated by arg min ℓ_1_(***x***, ***o***^(*k*)^). That is, the multivariate i.i.d. Bernoulli likelihood maximization problem can be approximately reduced to the minimization of the *L*_1_ discrepancy between the binary input and output of the VAE.

## Results

### Compression factor with VAE improves over PCA-based approach

Like many natural signals, SNP sequences can be viewed as realizations of a stochastic process. Such data exhibit a particularly high level of redundancy and correlation, owing in part to LD. For that reason, we have conducted a comprehensive entropy analysis to understand the statistical nature of SNP sequences and to motivate how population-specific genetic diversity influences compression performance, thereby connecting these observations to the main theme of efficient genomic data modeling with VAE. A detailed description of the employed data sets can be found in Supplemental Methods S4.

**Figure 1.**
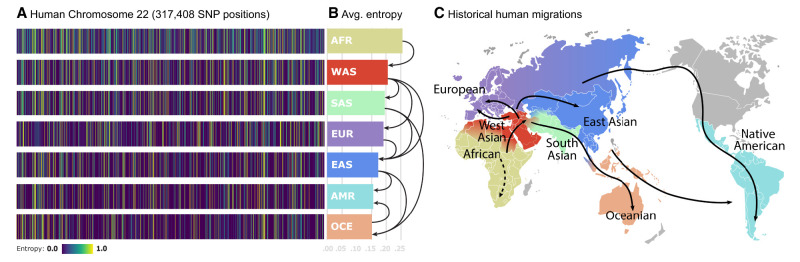
SNP entropy variation between populations. (*A*) We find 317,408 SNP entropies computed for human Chromosome 22 for each continental population. Observe that the uncertainty levels for particular SNP positions are different for each population. This is directly related to genetic variability. (*B*) Average of entropy vectors from *A*. Those values would correspond to estimated lower bounds of compression for each human population. Arrows represent the migration paths. (*C*) World map showing the main directions of human population migrations.

To estimate the entropies, we leverage the fact that for a Bernoulli random variable, its distribution parameter is given by the mean of the random variable. To avoid bias from the unbalancedness of the data set, we compute the entropy estimates per population by bootstrapping 32 samples from the founders’ pool 50 times and average them. The choice of 32 is significant as it is half of the size of the smallest human superpopulation in the data set, and the choice of 50 corresponds approximately to the fraction between the size of the largest human continental population divided by 32. The resulting density plots of the entropy rates for human Chromosome 22 for each superpopulation are depicted in Figure 1A. Among these populations, the African population (AFR) presents the highest variability in SNP values and, thus, highest entropy values per SNP. In contrast, the Oceanian (OCE) and Native American-like (AMR) populations exhibit the lowest values of entropy. These results align with the out of Africa (OOA) theory (Nielsen et al. 2017), suggesting that populations that migrated out of Africa experienced a reduction in genetic diversity and an increase in LD. Averaging the entropy vectors for each population yields the average SNP entropy per population, as shown in Figure 1B. These values signal an interesting connection to historical human migrations, as depicted in Figure 1C. Starting with African individuals, migrations led humans to expand to West Asia (WAS), South Asia (SAS), Europe (EUR), and East Asia (EAS). In these out-of-Africa regions, populations experienced a noteworthy reduction in average SNP entropy, reflecting a decrease in genetic diversity over time likely due to founder effects. As time progressed, subsequent migrations brought individuals to Oceania (OCE) and the Americas (AMR), culminating in populations with minimal genetic variability, as evidenced by their lower average SNP entropy. This observation aligns with the notion that genetic diversity tends to decrease as populations migrate further away from their ancestral origins and undergo genetic bottlenecks.

Based on the VAE architecture, the decoder obtains the reconstruction $\hat{x}$, which is a lossy representation of ***x***. To achieve lossless compression, it is necessary to store the residual, which is the elementwise difference between the input and the reconstruction, denoted as $r=|x-\hat{x}|$, with $r,x,\hat{x}\in B^{d}$. This residual is used for error correction of the reconstruction, and with a high-generalization level, a well-trained VAE can produce a sufficiently sparse ***r***. This sparsity can be compressed, in its turn, with another lossless algorithm A. For the sake of simplicity, in our PCA-VAE comparison experiments, we use run-length encoding (RLE) for A. We consider a compression execution successful when the inequality from Equation 8 holds:

(8) ℓ(z)+ℓ(A(r))<ℓ(x),

where ℓ(·) is the *size function*, which computes the number of digits of the binary representation according to the data type. The latent vector ***z*** is composed of floating-point latent factors, whereas the reconstructed bits can be stored as Booleans, which, in the best case scenario, should be sparse enough to enable compression with RLE into a smaller integer array.

Table 1 presents the results of human SNP compression for sequences of length of 10,000 SNPs, comparing PCA versus VAE models trained genome-wide. These models were fit to single-ancestry simulated data with 400 generations from founders with 100 individuals in each generation. As observed, VAEs with a bottleneck dimensionality of 32, 64, and 128 latent factors are capable of compressing all populations, including the African population (AFR) which exhibits the highest degree of variability. Notably, European (EUR) and Native American-like (AMR) ancestries can be compressed to half their original size. In contrast, PCA, being a linear method, struggles to reconstruct effectively from ***z***, resulting in a residual vector ***r*** that is not sufficiently sparse. Consequently, PCA leads to an expansion in size by factors of 2×, 3×, and 4×, rather than achieving compression. The VAE takes advantage of the nonlinearities in the decoder for improved reconstruction. We also explored compression using a C-VAE (Supplemental Results S1 and Supplemental Table S3).

**Table 1.** Compression factors of PCA versus VAE

Models			Populations						
Type	|*z*|	α	European (EUR)	East Asian (EAS)	Native American (AMR)	South Asian (SAS)	African (AFR)	Oceanian (OCE)	West Asian (WAS)
PCA	2	–	×0.41	×0.38	×0.42	×0.39	×0.33	×0.36	×0.38
VAE		10^−4^	×0.68	×0.64	×0.76	×0.62	×0.53	×0.50	×0.67
		10^−5^	×0.65	×0.61	×0.73	×0.60	×0.52	×0.49	×0.63
PCA	4	–	×0.41	×0.37	×0.41	×0.38	×0.33	×0.36	×0.38
VAE		10^−4^	×0.77	×0.69	×0.87	×0.68	×0.56	×0.58	×0.71
		10^−5^	×0.73	×0.69	×0.81	×0.66	×0.54	×0.63	×0.68
PCA	8	–	×0.41	×0.37	×0.41	×0.38	×0.33	×0.36	×0.37
VAE		10^−4^	×1.00	×0.93	**×1.17**	×0.88	×0.63	×0.68	×0.89
		10^−5^	×0.96	×0.90	**×1.08**	×0.88	×0.63	×0.74	×0.88
PCA	16	–	×0.40	×0.36	×0.41	×0.38	×0.32	×0.35	×0.37
VAE		10^−4^	**×1.59**	**×1.39**	**×1.73**	**×1.32**	×0.84	**×1.01**	**×1.37**
		10^−5^	**×1.25**	**×1.12**	**×1.35**	**×1.04**	×0.70	×0.90	**×1.08**
PCA	**32**	–	×0.39	×0.36	×0.40	×0.37	×0.32	×0.34	×0.36
**VAE**		**10** ^**−4**^	**×2.00**	**×1.75**	**×2.33**	**×1.69**	**×1.03**	**×1.27**	**×1.72**
		10^−5^	**×1.67**	**×1.45**	**×1.85**	**×1.39**	**×1.85**	**×1.23**	**×1.28**
PCA	**64**	–	×0.37	×0.34	×0.38	×0.35	×0.31	×0.33	×0.34
**VAE**		**10** ^**−4**^	**×2.04**	**×1.82**	**×2.27**	**×1.75**	**×1.16**	**×1.47**	**×1.82**
		10^−5^	**×1.69**	**×1.54**	**×1.96**	**×1.47**	×0.96	**×1.30**	**×1.49**
PCA	**128**	–	×0.34	×0.32	×0.35	×0.32	×0.29	×0.30	×0.32
**VAE**		**10** ^**−4**^	**×1.54**	**×1.45**	**×1.61**	**×1.41**	**×1.06**	**×1.25**	**×1.43**
		10^−5^	**×1.47**	**×1.37**	**×1.56**	**×1.35**	×0.97	**×1.20**	**×1.37**
PCA	256	–	×0.30	×0.28	×0.30	×0.28	×0.25	×0.27	×0.28
VAE		10^−4^	×0.97	×0.93	×1.00	×0.93	×0.79	×0.86	×0.93
		10^−5^	×0.94	×0.90	×0.97	×0.89	×0.73	×0.84	×0.90
PCA	512	–	×0.24	×0.23	×0.24	×0.23	×0.21	×0.22	×0.23
VAE		10^−4^	×0.54	×0.53	×0.55	×0.53	×0.48	×0.50	×0.53
		10^−5^	×0.53	×0.52	×0.54	×0.51	×0.45	×0.49	×0.52

The compression factors are computed as ℓ(x)/(ℓ(z)+ℓ(A(r))) using test data. A compression ratio of 1 corresponds to the identity, and values <1 and >1 correspond to compression and expansion, respectively. VAEs with a bottleneck of 32, 64, and 128 latent factors are capable of lossless compression of all human populations. Successful compression is marked in bold. |***z***| is the number of latent factors and α stands for *learning rate*.

### Leveraging autoencoders for lossless SNP compression in practice

**Figure 2.**
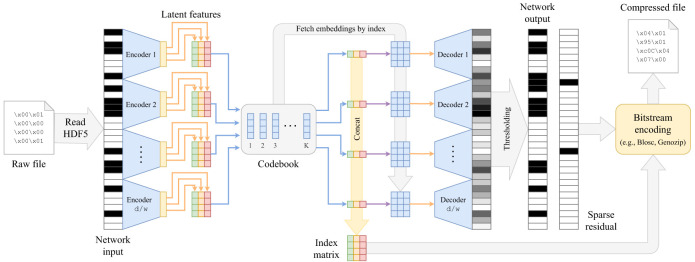
Proposed VQ-VAE architecture for genotype compression. The window-based VQ-VAE autoencoder processes an input SNP sequence ***x*** and encodes with $V_{e}(\cdot )$ into *H* bottleneck representations (*H* is the number of heads in the encoder). The quantizer Q substitutes the bottleneck representations by the closest codebook embeddings. Finally, the latent representation can be encoded as an integer index matrix. For the decoding step, codebook embeddings are fetched according to the indices of the index matrix and decoded as usual with the window-based autoencoder. The output is thresholded to obtain the reconstruction. The difference of the input with the reconstruction yields the residual ***r*** which, together with the index matrix, can be integrated in any bitstream-coding-based compression pipeline, such as Genozip (Lan et al. 2021), Zstandard (Collet and Kucherawy 2018), or Blosc (https://www.blosc.org).

The presented results inspire us to push even further the limits of genotype compression. Whereas previously we have used RLE for residuals, in practical production settings, we should transition to more efficient coding methods. Additionally, recognizing that a discrete representation often consumes less memory than a continuous one, we explore the concept of vector quantization within the autoencoder framework, known as VQ-VAE (van den Oord et al. 2018). We show that introducing the autoencoder into existing compression pipelines, such as Genozip (Lan et al. 2021) or Lempel–Ziv codecs (Collet and Kucherawy 2018; http://www.blosc.org), can lead to significant improvements in compression factors for large SNP data sets (Fig. 2).

To discretize the latent representation of ***x***, we employ a quantizer denoted as Q. This quantizer represents ***x*** as a matrix of positive integer indices, which point to a specific set of embeddings, as described in van den Oord et al. (2018). Formally, $(Q\circ V_{e})\;:\;B^{d}⟶Z_{+}^{H\times \lceil d/w\rceil }$, where *d* is the input dimensionality, *w* is the window size, and *H* corresponds to the number of heads in each window-encoder. With a uniform prior, the latent representation is defined by indices pointing to a fixed set of embeddings. We define the codebook size as *K* × *q*, where *K* is the number of embeddings, and *q* signifies the bottleneck dimensionality. We opt for utilizing multihead encoders with *H* heads. The rationale behind incorporating multiple heads is that the number of potential representations is determined by *K*^*H*^. By choosing *H* = 1, we would limit the possible representations to *K* embeddings, which might lead to different inputs mapping to the same quantized latent vector, resulting in nonsparse residuals.

In the context of discrete-space autoencoders, it is important to note that the Q operator is not differentiable. To address this limitation, we employ the straight-through gradient estimator, which allows for gradient flow during backpropagation. Additionally, within the VQ-objective, we introduce two additional loss terms: (a) the *embedding loss*, which encourages the embeddings to align with the encoder outputs, and (b) the *commitment loss*, which incentivizes the encoder to output ***z*** closer to the codebook embeddings ***e***_*k*_, 1 ≤ *k* ≤ *K*, where *K* is the size of the codebook and *ξ* denotes the stop-gradient operator:

(9) $$\begin{array}{rl} L_{\mathrm{VQ}}(x,o) & =L_{\mathrm{BCE}}(x,o)\\ & \;\;+\underset{\mathrm{Embedding}\;\mathrm{loss}}{\underbrace{\|\xi [z]-e_{k}{\|}_{2}^{2}}}+\underset{\mathrm{Commitment}\;\mathrm{loss}}{\underbrace{\|z-\xi [e_{k}]{\|}_{2}^{2}}} \end{array}.$$

The stop-gradient operator *ξ* acts as the identity during forward computation and has zero partial derivatives during backpropagation, treating its operand as a nonupdated constant (van den Oord et al. 2018), to avoid collapsing the optimization process because ***z*** and ***e***_*k*_ are mutually related, and both need to be optimized.

**Figure 3.**
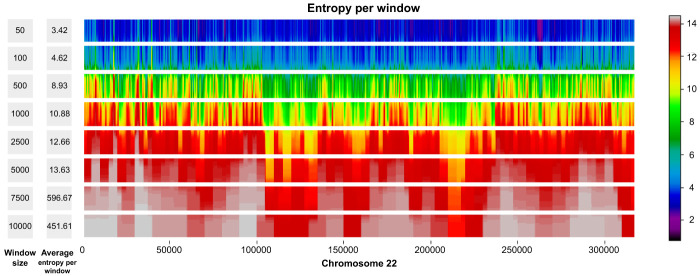
Average entropy per window across different window sizes on Chromosome 22. For each window size *w* ∈ {50, 100, 500, 1000, 2500, 5000, 7500, 10,000}, we compute the average entropy per window over the entire chromosome.

For production purposes, we consider window-based autoencoders with nonoverlapping windows. These autoencoders have two main hyperparameters: the window size *w* and the bottleneck size *b* for each window. Therefore, the index matrix maintains a fixed size of H×⌈d/w⌉. In our window-based architecture, an important decision revolved around the selection of values for *w* and *b*. A larger value for *b* results in more information being compressed into the latent representation, leading to improved reconstruction of ***x*** and consequently a sparser residual. A sparser residual can be better encoded with a coding algorithm A because of the longer homogeneous regions of zeros. Concerning the choice of *w*, we performed a heuristic analysis to identify an appropriate window size, settling on *w* = 2500. We first calculate the *average intrawindow entropy* (which depends on the number of unique sequences in a window) for each candidate window size in the set *w* ∈ {50, 100, 500, 1000, 2500, 5000, 7500, 10,000}. This yields a list of window sizes ***w*** = [*w*_1_, *w*_2_, …, *w*_*n*_] and a corresponding list of average intrawindow entropies across human Chromosome 22 for each choice (see Fig. 3), ***E*** = [*E*_1_, *E*_2_, …, *E*_*n*_]. Our rationale is that smaller windows would require a larger bottleneck to represent at least the same amount of information. For instance, if a binary sequence has length 18, using *w* = 3, the smallest possible bottleneck is going to be of size 6, whereas using *w* = 6, the smallest possible bottleneck is going to be of size 3. Consequently, there is an inherent trade-off between window size and the complexity needed in the model. To formalize our selection, we computed the difference in consecutive window sizes: Δ*w*_*i*_ = *w*_*i*+1_ − *w*_*i*_ and the difference in consecutive intrawindow entropies: Δ*E*_*i*_ = *E*_*i*+1_ − *E*_*i*_. For each interval *i*, we combine Δ*w*_*i*_ and Δ*E*_*i*_ (e.g., via their product, Δ*w*_*i*_ × Δ*E*_*i*_) to gauge how *much* extra entropy is gained when the window size changes from *w*_*i*_ to *w*_*i*+1_, and we look for the index *i* that maximizes this combined metric. Practically, this represents an “elbow,” beyond which increasing the window size further does not substantially alter the average intrawindow entropy. Thus, we aim to strike a balance between *model simplicity* (not using an overly large window size, which might lose fine-grained structure) and *encoding efficiency* (not using an excessively small window size, which would require a large bottleneck to capture all local information). In our analysis, *w* = 2500 results in a good compromise between capturing local genomic structure and maintaining a reasonable model bottleneck size.

The window-based encoder $V_{e}$ takes as input a SNP sequence ***x*** of a specific length *d* and compresses it to the dimensionality of the bottleneck *q* = *d*/*w* · *b*. In this manner, we obtain the vector of latent factors ***z***, which in its turn is quantized with Q. Next, the decoder $V_{d}$ reconstructs the input with some errors, $\hat{x}=1_{1/2}(o)$, where ***o*** represents the network’s output. As before, storing the residual $r=|x-\hat{x}|$ allows for a lossless compression of ***x***, as the reconstruction errors can be corrected using the residual. With a sufficiently large *q*, the residual ***r*** becomes sparse, making it amenable to compression. This sparsity can be compressed further with a bitstream coding lossless algorithm A, along with the latent representation ***z***. The celebrated Lempel–Ziv algorithm belongs to the class of universal compression schemes, and in our experiments, we use its variations for the role of A.

In this case, we consider a compression execution successful when Equation 10 holds (note the difference with Eq. 8):

(10) $$\ell (A(z))+\ell (A(|x-\hat{x}|))=\ell (A(z))+\ell (A(r))<\ell (x).$$

In the proposed method (Fig. 2), the window-based VQ-VAE autoencoder is composed of three hidden layers. Each fully connected layer is followed by a batch normalization layer (Ioffe and Szegedy 2015). The activation preceding the bottleneck is transformed by means of the hyperbolic tangent function, to a range which is useful for quantization in the context of discrete latent spaces. The activation at the output of the network is a sigmoid, which converts the activations into Bernoulli probabilities. All the other activation units in intermediate layers are ReLUs. At the beginning of the training process, all the weights and biases are initialized with Xavier initialization (Glorot and Bengio 2010). We employ the Adam optimizer (Kingma and Ba 2017) with the best-performing learning rate of α = 0.025 and a weight decay of γ = 0.01. A scheduler has been set to reduce the learning rate by a factor of γ = 0.1 in the event of learning stagnation. Furthermore, a dropout (Srivastava et al. 2014) of 50% has been introduced in all layers because it offers a better generalization providing larger compression factors (Supplemental Methods S8).

We benchmark the performance of our autoencoder + bitstream coding compression strategy against several compression methods, namely: Gzip (general-purpose), ZPAQ (Mahoney 2005) (for text), Zstandard (Collet and Kucherawy 2018; https://www.blosc.org) (general-purpose), bref3 (Browning et al. 2018) (for VCF), and Genozip (Lan et al. 2021) (general-purpose optimized for genomic data). We evaluate the compression on four different test sets, each containing 11,772 simulated individuals generated using Wright–Fisher simulation. These test sets consisted of HDF5 files with different numbers of SNPs from human Chromosome 22: 10,000 SNPs, 50,000 SNPs, 80,000 SNPs, and the entirety of human Chromosome 22 (317,400 SNPs). For compression with bref3 (Browning et al. 2018), we had to run an additional preprocessing step which would convert the HDF5 into a VCF file (the conversion runtime is not included in the benchmark). The results of this benchmark are summarized in Table 2, highlighting the advantages of incorporating autoencoders within compression pipelines.

**Table 2.** Compression benchmark for subsets of SNPs of human Chromosome 22

Data	10,000 SNPs	50,000 SNPs	80,000 SNPs	317,400 SNPs
Original size	112.27	561.33	898.14	3536.40
Gzip (clevel 9)	6.48 (×17.3) [1 m 0.691 s]	40.68 (×13.8) [6 m 9.370 s]	65.20 (×13.8) [10 m 45.854 s]	263.30 (×13.4) [44 m 5.341 s]
ZPAQ (clevel 3) (Mahoney 2005)	5.92 (×18.9) [1 m 59.611s]	28.83 (×19.5) [9 m 52.687 s]	45.18 (×19.9) [24 m 39.143 s]	183.38 (×19.3) [98 m 46.042 s]
Zstandard (Collet and Kucherawy 2018)	11.29 (×9.9) [0 m 0.209 s]	57.08 (×9.8) [0 m 1.017 s]	92.75 (×9.7) [0 m 2.143 s]	372.74 (×9.5) [0 m 6.535 s]
Genozip (Lan et al. 2021)	**0.94 (×119.4)** [0 m 12.899 s]	29.89 (×18.8) [0 m 2.681 s]	48.67 (×18.5) [0 m 3.249 s]	200.13 (×17.7) [0 m 11.741 s]
bref3 (Browning et al. 2018)	4.35 (×25.8) [0 m 1.383 s]	**19.91 (×28.2)** [0 m 4.322 s]	**27.31 (×32.9)** [0 m 10.709 s]	**115.52 (×30.6)** [0 m 22.916 s]
VQ-VAE + Zstandard (ours)	**3.42 (×32.83)** [0 m 12.905 s]	25.37 (×22.12) [1 m 0.564 s]	40.17 (×22.4) [1 m 42.669 s]	160.68 (×22.0) [6 m 37.681 s]
VQ-VAE + Genozip (ours)	3.59 (×31.3) [0 m 6.984 s]	**19.44 (×28.9)** [0 m 14.447 s]	**27.77 (×32.3)** [0 m 26.471 s]	**115.24 (×30.7)** [1 m 23.828 s]

The file size in MB is compared between methods, along with its compression factor and running time. We mark in bold the top two choices based on compression factors.

### Dimensionality reduction with VAE

Genotypes can unravel population structure. The identification of genetic clusters can be important when performing GWAS and provides an alternative to self-reported ethnic labels, which are culturally constructed and vary according to the location and individual. A variety of unsupervised dimensionality reduction methods have been explored in the past for such applications, including PCA, MDS, t-SNE (Van der Maaten and Hinton 2008), and UMAP (McInnes et al. 2018). Recently, VAEs have been introduced into population structure visualization (Battey et al. 2021; Meisner and Albrechtsen 2022). Battey et al. 2021). The singular feature of VAEs is that they can represent the population structure as a Gaussian-distributed continuous multidimensional representation and as classification probabilities providing flexible and interpretable population descriptors. Besides, latent maps allow for meaningful interpretation of distances between ancestry groups. Although it is true that proximity in the latent space cannot be directly interpreted as proportional to similarity—a recurrent issue highlighted in nonlinear dimensionality reduction techniques, such as t-SNE and UMAP (Battey et al. 2021; Chari and Pachter 2023) but also present in PCA (Elhaik 2022; Montserrat and Ioannidis 2023)—the implicit regularization of the optimization process of VAEs and the capacity of the encoder/decoder can limit the distortion on the local and global distances and, as observed experimentally, such projections can still provide insights in the structure of the data which are discussed next.

**Figure 4.**
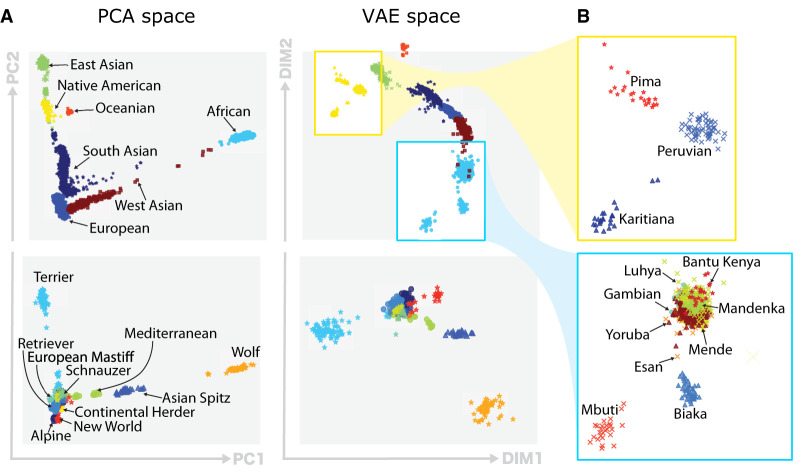
Qualitative comparison of PCA and VAE projections. (*A*) The *top* row illustrates the projections generated by both PCA and VAE for 4894 human samples using 839,629 SNPs. The *second* row displays projections of 489 canine samples using 198,473 SNP positions. (*B*) Focus of VAE projections of Native American-like subpopulations (in yellow) and African subpopulations (in blue).

We quantitatively assess the quality of the VAE clusters by comparing the clustering performance of PCA and VAE clusters. We use the pseudo *F* statistic (Caliński and Harabasz 1974), the Davies–Bouldin index (DBI) (Davies and Bouldin 1979), and the silhouette coefficient (SC) (Kaufman and Rousseeuw 2009) as clustering metrics (Supplemental Methods S7). We use two principal components to cluster populations, and although two might be a small number of components for data explainability, the quantitative (Table 3) and qualitative (Fig. 4) results show that two VAE components retain more information than two PCA components.

**Table 3.** Comparison of clustering performance of PCA versus VAE

	Data type	Human data	Canine data
Pseudo *F* statistic	PCA	50,315.56	151.89
	VAE	**56,409.29**	**276.03**
Silhouette coefficient	PCA	0.69	**0.12**
	VAE	**0.77**	0.07
Davies–Bouldin index	PCA	0.48	3.87
	VAE	**0.29**	**3.39**

PCA and VAE parameters have been fitted to human and canine SNP data sets of 839,629 and 198,473 SNP positions, respectively. Clustering metrics have been computed on seven self-reported human ancestry groups and 16 canine clades composed of 144 distinct canine breeds. The 2D latent coordinates of the samples have been standardized. Bold values indicate the better-performing method for each metric and data type (higher is better for Pseudo *F* and Silhouette; lower is better for Davies–Bouldin).

Visually, the VAE clusters still preserve the geographic vicinity of adjacent human populations and, additionally, discriminate more than PCA some subpopulations within each cluster, as it can be clearly observed in the case of African (AFR) and Native American-like (AMR) populations in Figure 4B. Therefore, VAE projections to the two-dimensional space allow for a more insightful and fine-grained exploratory analysis. As an example, having a closer look to the aforementioned ancestry groups, shown in Figure 4B, observe that PCA is not capable of differentiating their subpopulations. In contrast, VAE clusters in the African population clearly distinguish Mbuti and Biaka subpopulations, which both are hunter gatherer populations from the central African cluster (Supplemental Figs. S3–S5). Another interesting visualization is the projection of canine genotypes to the VAE latent space (Fig. 4A). For instance, the Asian Spitz clade is found closer to the wolves, which suggests their genetic similarity as they were one of the first domesticated canids (Yang et al. 2017). The list of human and canine populations can be found in Supplemental Tables S1 and S2.

Regarding other nonlinear manifold learning techniques, such as t-SNE (Van der Maaten and Hinton 2008) and UMAP (McInnes et al. 2018), these are not directly comparable with our approach as they do not allow mapping new samples to the spanned space, which makes them not usable for the tasks of compression, classification, and simulation. The reason is that both methods learn a nonparametric mapping; that is, they do not learn an explicit function that maps data from the input space to the map (Supplemental Fig. S6). Therefore, it is not possible to embed test points in an existing map.

### Different objectives for ancestry classification

**Figure 5.**
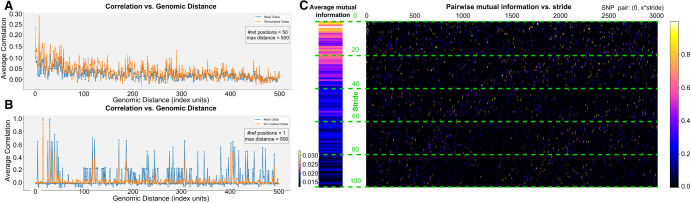
Metrics for assessing LD structure and inter-SNP correlation. (*A*) Average correlation versus genomic distance for real (blue) and simulated (orange) genotypes. We select multiple reference SNPs and plot the correlation with neighboring positions up to specified maximum distances (in the example, we use a distance of 500 with 50 reference positions). (*B*) Correlation of a single SNP position, illustrating how well the synthetic data reproduce the LD structure. (*C*) Pairwise mutual information between a reference SNP and other SNPs (*x*-axis) separated by varying stride lengths (*y*-axis), so that the resulting value corresponds to the SNP pair (0, *x* · *y*). The color scale indicates the magnitude of mutual information, from negligible (dark) to higher (bright) values.

Individuals that are more closely related in ancestry have spatial autocorrelations in their genomic sequences. This phenomenon is translated into population clusters using dimensionality reduction techniques (Fig. 4). The most common approaches to address ancestry classification and regression are based on dimensionality reduction and clustering techniques (Tan and Atkinson 2023). Genomic sequences from known and unknown origins are jointly analyzed—unknown samples are assigned to the nearest labeled cluster of the feature space (e.g., PCA space). Yet there are some caveats (Baran et al. 2013; Battey et al. 2020): (a) The results can be nonsensical if the individuals to classify are admixed, that is, are descendants of individuals from different ancestries, or do not originate from any of the sampled reference populations (*out-of-sample*); (b) commonly used dimensionality reduction techniques such as PCA do not model LD. Correlations induced by LD violate the inherent assumptions of independency between SNP positions. The cumulative effect of those correlations not only decreases accuracy but can also bias the results, a fact that can be observed in PCA projections of sequential SNP positions compared to SNP positions selected at random (Supplemental Figs. S7 and S8). In contrast, VAE, the nonlinear counterpart of PCA, is able to model up to a certain degree these induced correlations (see Fig. 5B) allowing for less LD bias with a relatively small number of SNP positions as input and consequently, yielding better visualizations.

For the classification task, we have trained VAEs on 10,000 sequential SNP positions from human Chromosome 22. The learned representations with VAEs have been assessed based on the population labels we have for each individual. We provide a quantitative evaluation of these learned representations using different classification approaches which are described in detail next.

The simplest idea for classification in the latent space is computing the population centroids and assigning each individual to the nearest one. We refer to this method as the *nearest latent centroid* heuristic. It is a form of nearest-neighbor classification based on the latent features extracted by the VAE. Each centroid $c_{k},1\leq k\leq |Y|$, is computed as

(11) $$c_{k}=\frac{\sum_{n=1}^{N}1_{k}(x_{n})z_{n}}{\sum_{n=1}^{N}1_{k}(x_{n})},$$

where $z_{n}=V_{e}(x_{n})$ and $1_{k}(\cdot )$ is an indicator function that denotes membership to ancestry label *k*. For each ***x***_*n*_, we assign the label that minimizes the distance between the latent representation and the corresponding centroid:

(12) $$y_{n}=\begin{array}{c} \arg\;\min\\ k\in Y \end{array}\|z_{n}-c_{k}{\|}^{2}.$$

In the classification results presented in this study, we set |Y|=7 as we have seven human superpopulations (continental) groups in the provided data set. We hypothesized that each ancestry-conditioned VAE would better reconstruct the population to which it belongs. In the case of Y-VAE, each independent VAE learns to reconstruct better the population on which it has been trained, which is translated into the minimization of the *L*_1_ norm between the input SNP array and the reconstruction. Let us denote the composition of encoder $V_{e}^{\left(k\right)}(\cdot )$, decoder $V_{d}^{\left(k\right)}(\cdot )$, and binarization $1_{1/2}(\cdot )$ functions, as $f_{\theta }^{\left(k\right)}(\cdot )$ to which we refer to as the VAE model conditioned on *k*th ancestry with parameters ***θ***. Then, the ancestry of the *n*th sample:

(13) $$\begin{array}{rl} y_{n} & =\underset{k\in Y}{\arg\;\min}\|x_{n}-{\hat{x}}_{n}^{\left(k\right)}{\|}_{1}\\ & =\underset{k\in Y}{\arg\;\min}\|x_{n}-f_{\theta }^{\left(k\right)}(x_{n}){\|}_{1} \end{array}.$$

With such conditioning, a Bayesian parameter estimation approach can be adopted for ancestry label inference via MAP estimation. Refer to the “Methods” section for the complete mathematical derivation.

The encoder–decoder architecture, when conditioned on *k*th ancestry, produces a vector of Bernoulli probabilities $o_{n}^{\left(k\right)}$, which is subsequently thresholded to generate ${\hat{x}}_{n}^{\left(k\right)}=1_{1/2}(o_{n}^{\left(k\right)})$. By training the network with the BCE loss, we assume that each individual SNP position *x*_*ni*_, 1 ≤ *i* ≤ *d*, follows a Bernoulli distribution. This assumption allows us, in theory, to calculate the Bernoulli likelihood for each SNP position. A MAP estimate of *Y* is the one that maximizes the posterior probability *p*(*Y* = *k*|***x***_*n*_) for a given sample ***x***_*n*_, which is given by

(14) $$\begin{array}{rl} y_{n} & =\underset{k\in Y}{\arg\;\min}p(Y=k\;|\;x_{n})\\ & \;\propto \underset{k\in Y}{\arg\;\min}\sum_{i=1}^{d}x_{ni}\log o_{ni}^{\left(k\right)}+(1-x_{ni})\log\left(1-o_{ni}^{\left(k\right)}\right)\\ & \;=\arg\;\min_{k\in Y}\ell_{\mathrm{BCE}}(x_{n},o_{n}^{\left(k\right)}). \end{array}$$

Note that the BCE minimization problem can be approximated by the minimization of the *L*_1_ discrepancy between the input and the output of the VAE. Refer to the “Methods” section for details.

To conclude the classification methods section, we present the results for each method in Table 4. The first two rows compare the *nearest latent centroid* approach in PCA and VAE spaces. Notably, there is a substantial improvement when using the VAE-generated space instead of PCA. The overall test accuracy increases from 74.1% to 85.7%, representing a >15% increase in accuracy. Both C-VAE and Y-VAE are evaluated based on two criteria: the minimization of the discrepancy between the input and the reconstruction, and the maximization of the Bernoulli likelihood. Among these, C-VAE, which maximizes the BCE loss, demonstrates the best performance across all populations, achieving an accuracy of 87.1% on the test data. It is worth noting that Oceanian (OCE) and West Asian (WAS) populations exhibit the lowest classification accuracy. Coincidentally, those two populations had the smallest number of founders for simulation. One possible explanation for this phenomenon is that the variability within the simulated samples is insufficient to provide robust generalization for the VAE, necessitating a larger number of founders for improved performance.

**Table 4.** Accuracy of classification methods

Model	Criterion	All		EUR		EAS		AMR		SAS		AFR		OCE		WAS	
		TR	TS	TR	TS	TR	TS	TR	TS	TR	TS	TR	TS	TR	TS	TR	TS
PCA	${\arg\;\min}_{k}\|z_{n}-c_{k}{\|}_{2}^{2}$	78.6	74.1	64.9	66.3	71.1	74.3	87.2	77.8	58.5	57.2	97.5	93.4	95.4	76.6	75.6	73.4
VAE		93.2	85.7	81.7	78.6	96.9	96.3	99.5	92.1	81.6	78.5	99.3	96.8	99.4	71.7	94.0	**86.1**
C-VAE	${\arg\;\min}_{k}\|x_{n}-{\hat{x}}_{n}^{\left(k\right)}{\|}_{1}$	93.4	78.0	84.4	70.6	96.4	92.1	99.9	92.4	84.4	76.4	99.9	96.8	100	62.8	88.5	54.7
	arg max_*k*_ *p*(*Y* = *k*|***x*_*n*_**, ***θ***)	97.5	**87.1**	96.4	**87.1**	98.5	95.5	100	**97.4**	90.0	81.2	99.4	94.9	100	**79.8**	98.1	73.5
*Y*-VAE	${\arg\;\min}_{k}\|x_{n}-{\hat{x}}_{n}^{\left(k\right)}{\|}_{1}$	98.9	83.2	96.9	81.5	99.6	96.2	99.9	87.2	98.5	**90.1**	100	**98.4**	100	68.6	97.5	59.9
	arg max_*k*_ *p*(*Y* = *k*|***x*_*n*_**, ***θ***)	99.1	85.2	97.6	84.3	99.7	**96.6**	100	90.9	98.2	88.5	100	98.2	100	72.7	98.3	65.0

TR refers to accuracy computed on training data and TS on test data, accordingly. The values represent the accuracy in %. Note that regular VAE, C-VAE, and Y-VAE have 10,371,760, 10,378,928, and 72,602,320 parameters, respectively. Bold values indicate the highest accuracy (best performance) on test samples across the compared models and criteria.

### Synthetic data generated by VAE

**Figure 6.**
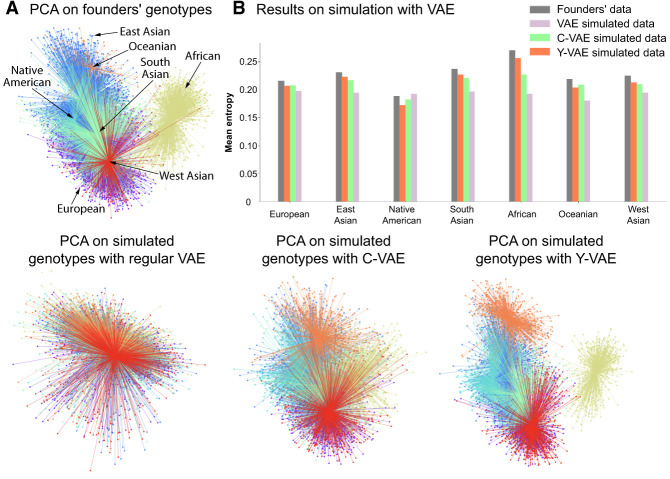
PCA of simulated genotypes and entropy comparison. The number of simulated samples is equal to the number of founders. (*A*) The *top-left* plots display the PCA projection of founders’ genotypes. The plots at the *bottom* line display PCA projections of synthetic genotypes simulated with regular VAE, C-VAE, and Y-VAE, in that order. (*B*) The method that best approximates the entropy distribution is Y-VAE. The least effective method is without conditioning, because the entropy is approximately the same across all populations.

Generating synthetic data with VAE methods is reasonably straightforward: one *could* simply sample z∼N(0,1) and decode to generate synthetic SNP sequences. However, our goal is to provide a mechanism for producing samples *specific to a given ancestry*. In the initial approach, a regular VAE (without conditioning) has been used to compute the population-specific centroids and variances in learned latent space, ***μ*** and ***σ***^2^, respectively. With these central points for each cluster, we sample from the isotropic multivariate Gaussian distribution, $N(\mu ,\sigma^{2}I)$, and decode the resulting latent vector with the VAE decoder $V_{d}(\cdot )$. However, there is no inherent reason to assume each population forms a Gaussian cluster and this approach does not yield distinct clusters because the distances between the centroids are not sufficiently large to differentiate between populations (Fig. 6A). Based on the insights gained from our simulation experiments, we have recognized that explicit conditioning is essential. The refined simulation algorithm involves sampling a multivariate Gaussian vector, z∼N(0,1), and then conditioning the decoder $V_{d}^{\left(k\right)}(\cdot )$ to map this *q*-dimensional latent vector into a *d*-dimensional simulated SNP array $\hat{x}$ of *k*th ancestry. By passing ancestry labels directly to the decoder, we gain an explicit “handle” on which population’s genetic templates we want to express (Supplemental Methods S3 and Supplemental Fig. S10).

In order to quantitatively assess the quality of the simulated individuals, we employ population genetics metrics such as LD patterns among SNPs (correlation structures, see Fig. 5) and folded allele frequency spectra (histogram of SNPs at 1%, 2%,…up to 50%; Supplemental Fig. S9), and we also leverage the entropy measure, as previously described in Geleta et al. (2025). In what follows, we differentiate between random variables, for example, ***X***, and samples, for example, ***x***, using uppercase and lowercase letters, respectively. In a genotype array denoted as ***X*** = [*X*_1_, …, *X*_*d*_], each SNP *X*_*i*_, where 1 ≤ *i* ≤ *d*, is considered a random variable taking values in the Boolean domain B={0,1} with $P_{X_{i}}(x)$ representing the probability mass function for *X*_*i*_. SNPs are typically modeled using a Bernoulli distribution. The entropy of *X*_*i*_ is defined as

(15) $$H(X_{i}){|}_{X_{i}∼P_{X_{i}}}=-\sum_{x\in B}P_{X_{i}}(x)\log_{2}P_{X_{i}}(x).$$

We use the entropy to compute the mutual information for a pair of SNPs (*X*_*i*_, *X*_*j*_), which measures their mutual dependence and quantifies how much information one position provides about the other. Formally, it is defined as

(16) $$I(X_{i};X_{j})=\sum_{x_{i}\in B}\sum_{x_{j}\in B}P_{\left(X_{i},X_{j}\right)}(x_{i},x_{j})\log_{2}\left(\frac{P_{\left(X_{i},X_{j}\right)}(x_{i},x_{j})}{P_{X_{i}}(x_{i})P_{X_{j}}(x_{j})}\right).$$

Our analysis of pairwise mutual information (Fig. 5C) indicates that most pairs of SNPs exhibit negligible mutual information, which is consistent with the expectation that long-range LD is weak or absent due to recombination (Gravel 2012).

In Figure 5A and B, we contrast LD patterns between real and simulated samples, observing that the correlation between variants decays with distance. Notably, our VAE-generated SNP sequences capture locus-specific LD structure—evident in correlation peaks that align with those in real data. Because simulated samples of a specific ancestry should closely follow the distribution of their respective founders (the real genotype samples from our simulation pool), comparing the entropy of real and simulated SNPs provides a robust measure of divergence. Indeed, Figure 6B demonstrates that the best-performing simulation method (Y-VAE) produces an entropy distribution closely matching that of the founders, whereas the least effective method (unconditional VAE) results in an entropy distribution that is nearly uniform across populations. Additionally, Supplemental Results S2 and Supplemental Fig. S1 show that our synthetic genotypes support accurate downstream ancestry classification.

Finally, to contextualize VAE performance with another deep generative approach, we include a comparison to a generative moment matching network (GMMN), trained on the same subset (5000 SNPs from 10,000 samples) following Perera et al. (2022). Detailed ablations, computational constraints, and additional figures are provided in Supplemental Results S3 and Supplemental Fig. S2.

## Discussion

We have demonstrated the power of VAEs applied to genomic variation analysis, providing promising performance in a variety of applications. We have conducted both qualitative and quantitative assessments of the quality of VAE clusters on human and canine SNP data sets, reinforcing the benefits of VAE for dimensionality reduction in a population genetics context. Most notably, we have developed a novel VAE-based lossless compression system tailored for SNP data and demonstrated how it can be integrated into existing SNP data set compression pipelines. We have also benchmarked VAE-based global ancestry classification against PCA-based classification and proved that the nonlinear approach performs better. Because of the introduced nonlinearities, the method is less sensitive to correlations of SNPs due to LD, resulting in an increased ability to capture complex population structure and represent relatively good ancestry differentiation and, unlike previous fully connected approaches (Battey et al. 2021), our window-based approach has shown to capture LD. In contrast to previous work for genotype simulation with VAEs (Battey et al. 2021), we have used VAE conditioning, which we found essential for generating high-quality simulated SNP sequences. VAE simulation provides an efficient method for SNP data simulation—we have conducted an entropy study on SNP data and reaffirmed the hypothesized migration paths (Nielsen et al. 2017) and phylogenetic relationships of the population groups, along with the theoretical compression bounds based on the statistical nature of SNP data.

In the context of our discussion, it is imperative to acknowledge several limitations associated with VAEs. First, when VAEs are employed for applications in data interpretability or visualization, the nonlinearity of the model may give rise to potentially misleading insights, akin to the caveats encountered in various dimensionality reduction techniques (Battey et al. 2021; Chari and Pachter 2023; Montserrat and Ioannidis 2023). Second, models trained specifically for a single task in a supervised fashion (e.g., classification) can outperform VAEs in terms of accuracy, a pattern that has been observed in other multimodal generalist models (Tu et al. 2024), and thus, the adoption of those task-specific specialist models may be a better option in scenarios where precise task execution is the primary objective. Finally, an additional limitation arises when considering the widespread adoption of VAEs for genomic compression. VAEs trained on a specific set of SNPs may exhibit challenges when applied to genomic data encompassing different genetic positions, and an adaption to new genetic positions could require retraining the network.

## Code availability

The source code is publicly available at GitHub (https://github.com/AI-sandbox/aegen) and as Supplemental Code.

## Competing interest statement

A.G.I. and D.M.M. own shares in Galatea Bio, Inc. The remaining authors declare no competing interests.

## Supplemental Material

Supplement 1

Supplement 2
