# Supplementary figures and images for: Autoencoders for genomic variation analysis
Source: Genome Res. 2026 Feb;36(2):348–60. doi: 10.1101/gr.280086.124 (PMC12863191; doi:10.1101/gr.280086.124)

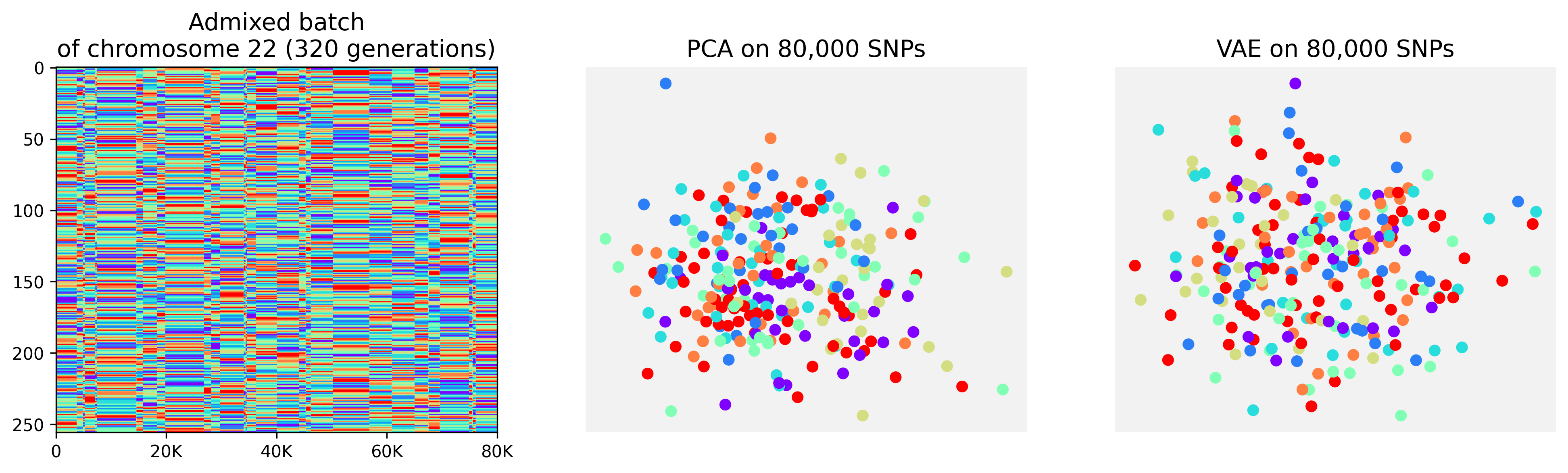

Supplement: Supplement 1 [file Supplemental_Code.zip › aegen-main/images/admixed_320_gens.png]

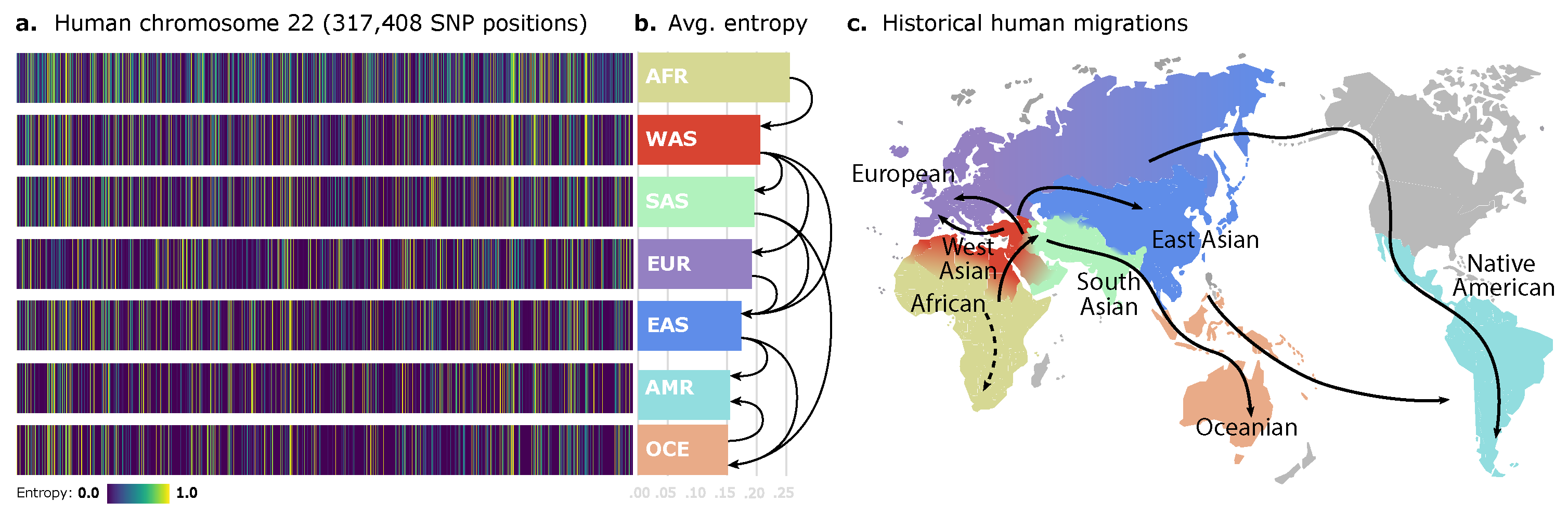

Supplement: Supplement 1 [file Supplemental_Code.zip › aegen-main/images/figure_3.png]

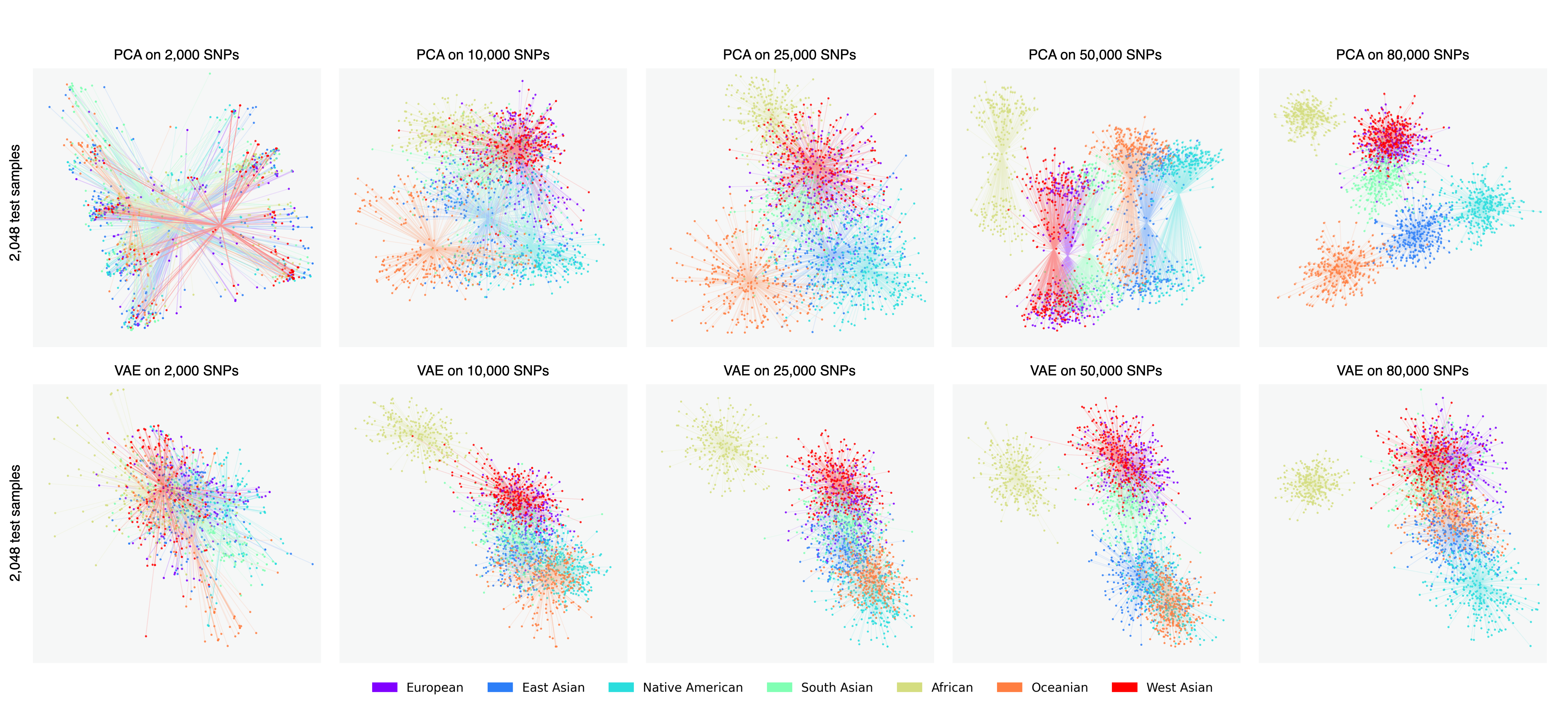

Supplement: Supplement 1 [file Supplemental_Code.zip › aegen-main/images/pca_vs_vae.png]
