# Supplementary material for: Autoencoders for genomic variation analysis
Source: Genome Res. 2026 Feb;36(2):348–60. doi: 10.1101/gr.280086.124 (PMC12863191; doi:10.1101/gr.280086.124)
Supplement: Supplement 2 [file Supplemental_Material.pdf]

Supplemental Material

Supplemental Methods S1. Relevance of this work for precision medicine

Pharmacogenetics, the study of the response of individuals to drug therapy based on their genome, is a key aspect in precision medicine applications. While external factors, such as diet or environment, can have influence on medication response, genomic information plays an important role, with different individuals having different responses to the same drug. A classic example is ibufenac's hepatotoxicity differing significantly between the UK and Japan (Shah 2013). Precision medicine aims to maximize the efficacy of drugs and mitigate the risks of side effects by mapping the right drug and the right dose to each individual with the help of genetic data (Shah and Gaedigk 2018). Because the genome determines the expression of all the organism's enzymes, including the ones that metabolize drugs, a drug response of an individual is directly dependent on the genetically determined concentration-response relationships of the enzymes. *Pharmacokinetics* (how an organism affects a drug) and *pharmacodynamics* (how a drug affects an organism) can have significant differences at both individual and population-level (Suarez-Kurtz 2008). To illustrate, in the US, as there is a great ancestral diversity in the overall population, some drug labels include clinical trials' data on diverse cohorts because of possible population differences in drug response. Therefore, the population differences in drug response have become crucial to establish public health policies, to design and evaluate clinical trials, and to develop, approve, and promote new drugs (Shah and Gaedigk 2018). A recent paper by Tempus Labs, Inc. (Rhead et al. 2023) also underscores the commercial and ethical significance of accurate ancestry inference to ensure equity in precision medicine.

Supplemental Methods S2. Rationale behind our choice for a VAE-based approach for SNP data modeling

In light of recent transformer-based approaches for genomic data, we clarify why we use an autoencoder-based method, rather than a self-attention architecture: **(a)** first of all, transformer-based models, such as gLM2 (Cornman et al. 2024), Nucleotide Transformer (Dalla-Torre et al. 2024), GPN-MSA (Benegas et al. 2025), or GROVER (Sanabria et al. 2024), are trained on base pairs, drawing their motivation from *large*

language models (LLMs) trained on natural language and they model nucleotide sequences as sentences and k-mers are words. By contrast, our setting involves binary SNP data (i.e., the presence or absence of a particular allele at a subset of genomic positions that vary). In essence, these are *sparse* and *tabular* features, not continuous or categorical token sequences. The benefits of LLMs for capturing long-range linguistic-like patterns do not directly extend to SNP genotype matrices. The main reasons are explained next; **(b)** SNP data is not translation invariant, i.e., changing a pattern of SNPs completely results in an alteration of semantic information. Because of this, SNP data is generally treated as tabular rather than sequential data. Moreover, as shown in Figure 5, SNPs do not exhibit long-range population-structure correlations. Our analysis of pairwise mutual information between SNP positions indicates that most pairs of SNPs (X_i, X_j) have negligible mutual information.

On top of that, in population genetics, principal component analysis (PCA) remains a strong baseline (Tan et al. 2023). Even more advanced methods for ancestry inference such as ADMIXTURE (Ali-Khan et al. 2010) (soft k-means) can be recast as an autoencoder-like technique.

Supplemental Methods S3. Options for simulating admixed individuals under the VAE model

Although our experiments in the main manuscript focus on simulating single-ancestry individuals, the conditional VAE framework readily permits simulating admixed genomes. For instance, with a C-VAE, one could partially weigh one-hot encodings for two or more populations. Alternatively, a Y-VAE could sample multiple latent embeddings for different ancestral groups and then combine them. See Supplemental Figure S10 for PCA versus VAE projections of admixed individuals.

Supplemental Methods S4. Datasets

We employ a human genome dataset comprising multiple sequences derived from publicly available human whole genome sequences collected from diverse world-wide populations. The three sources are: **(a)** *The 1000 Genomes Project* (The 1000 Genomes Project Consortium 2015), reporting genomes of 2,504 individuals from 26 populations from all continents; **(b)** *The Human Genome Diversity Project* (Bergström et al. 2020), adding 929 diverse genomes from 54 geographically, linguistically, and culturally diverse human populations; **(c)** *The Simons Genome Diversity Project* (Mallick et al. 2016), providing genomes from 300 individuals from 142 diverse populations. The dataset is pruned to contain only individuals identified as belonging to a single genetic ancestry cluster via ADMIXTURE unsupervised clustering without recent admixture. We use single-ancestry labels to simplify the interpretability of the clusters within the latent space, however, it is important to note that the method itself is not limited by the type of labeling used. After pruning, the dataset comprises 2,965 single-ancestry phased human genomes, each containing a maternal and paternal copy, resulting in a total of 5,930 haploid sequences, referred to as *founders*. Founders are split into three non-overlapping groups with proportions 80%, 10% and 10%, to generate the training, validation, and test sets, respectively. Following the simulation scheme outlined in Montserrat, Bustamante, et al. 2020, for each dataset, we simulate samples with the corresponding set of founders via online *Wright-Fisher* simulation (Gravel 2012; Maples et al. 2013), where, at each VAE forward step, the online simulator produces new samples on-the-fly for each population separately, basing the recombination on the human *HapMap* genetic map (The International HapMap Consortium 2005). In each simulated batch, we ensure an equal number of individuals are generated for each population group, preventing training bias toward any specific ancestry. Note that we allow admixture at the subpopulation level, except for the dimensionality reduction task, in which we simulate samples within each of the 55 human subpopulations disjointly, so that there is no admixture among subpopulations – thus revealing more granular structure within each population. Refer to the Supplemental Table S1 for the full subpopulations list.

The canine genotyping array dataset consists of 722 canine whole genome sequences sourced from Plassais et al. 2019. For this dataset, we sample the most variable SNP

positions among breeds across 38 canine chromosomes, which correspond to a subset of SNPs that matches the genotyping array used by Bartusiak et al. 2022. This dataset encompasses a diverse range of canids, documenting wild canids, indigenous and village dog populations, as well as 144 domestic dog breeds. To manage the extensive breed diversity, we group similar breeds into 15 distinct clades, as outlined in the Supplemental Table S2.

Supplemental Table S1. List of human populations in our dataset.

Groupings	Size	Groupings	Size	Groupings	Size	Groupings	Size
**African (AFR)**							
Yoruba	130	Biaka	22	Bantu South Africa	4	Masai	2
Gambian Mandinka	113	Mandenka	22	Dinka	3	Saharawi	2
Luhya	100	Mbuti	13	Bantu Tswana	2	Bantu Herero	2
Esan	99	Bantu Kenya	11	Khomani San	2	Somali 2	
Mende	85	San	6	Luo	2		
**Native American-like (AMR)**							
Peruvian	31	Surui	8	Mexican-American	4	Piapoco	2
Karitiana	12	Maya	7	Mixe	3	Zapotec	2
Pima	12	Colombian	4	Quechua	3	Chane	1
**East Asian (EAS)**							
Japanese	131	Yi	10	Mongolian	9	Ami	2
Southern Han Chinese	105	Uygur	10	Lahu	8	Igorot	2
Han Chinese	103	Daur	10	Naxi	8	Eskimo Sireniki	2
Kinh Vietnamese	99	Northern Han	10	Even	3	Eskimo Naukan	2
Dai Chinese	93	Tujia	9	Tubalar	2	Burmese	2
Han	33	Oroqen	9	Ulchi	2	Itelman	1
Yakut	25	Hezhen	9	Thai	2	Eskimo Chaplin	1
Miao	10	Dai	9	Korean	2	Atayal	1
She	10	Cambodian	9	Mansi	2	Altaian	1
Tu	10	Xibo	9	Kyrgyz	2		
**European (EUR)**							
Tuscan	115	Basque	23	Abkhasian	2	Norwegian	1
Spanish	107	Adygei	16	Hungarian	2	Czech	1
Finnish	99	Orcadian	15	Estonian	2	Polish	1
British	91	Bergamo Italian	12	Crete	2	Chechen	1
Sardinian	28	Icelandic	2	Bulgarian	2	Samaritan	1
French	28	Sámi	2	Greek	2	Albanian	1
Russian	25	North Ossetian	2	Lezgin	1		
**Oceanian (OCE)**							
Papuan	17	Bouganville	11	Australian	2	Dusun	2
**South Asian (SAS)**							
Gujarati	103	Makrani	25	Hazara	19	Kusunda	2
Indian Telugu	102	Balochi	24	Tajik	2	Kapu	2
Sri Lankan	102	Pathan	24	Relli	2	Irula	2
Punjabi	96	Burusho	24	Yadava	2	Brahmin	2
Bengali	86	Sindhi	24	Mala	2	Khonda Dora	1
Brahui	25	Kalash	22	Madiga	2		
**West Asian (WAS)**							
Bedouin	46	Mozabite	27	Georgian Mingrelian	2	Turkish Cappadocia	2
Palestinian	46	Jordanian	3	Iranian	2	Yemenite Jew	2
Druze	42	Armenian	2	Iraqi Jew	2	Lezgin	1

Supplemental Table S2. List of canine populations in our dataset.

Groupings	Size	Groupings	Size	Groupings	Size
Terrier	115	European Mastiff	29	Spaniel	17
Wolf	51	Continental Herder	26	Drover	16
Retriever	51	Alpine	22	Mediterranean	13
UK Rural	47	New World	20	Scent Hound	12
Asian Spitz	29	Poodle	18	Pointer Setter	11

Supplemental Methods S5. VAE architecture

Given the non-linear relationship between the observed and latent variables, autoencoders are exceptionally well-suited for modeling genomic sequences. They excel in the task of learning novel, meaningful, and compact representations of SNP sequences, which represent the most prevalent genetic variations. The proposed VAE consists of a highly-adaptable and modular architecture, designed to accommodate different modes by utilizing flags for conditioning and denoising. Additionally, the model accepts two sets of parameters: **(a)** a set of *fixed parameters*, which defines essential aspects such as the number and size of layers in the encoder/decoder, dropout, batch normalization and activation functions, and **(b)** a set of *hyperparameters*, which defines optimizer-related flags and values, including the learning rate α, variational β, weight decay γ, among others. The proposed VAE is composed of two symmetric MLP sub-networks: the encoder and the decoder. Both blocks consist of a stack of either fully-connected layers or windowed fully-connected layers – depending on the task, the network can split the input SNP sequence into fixed-size non-overlapping windows, similarly as in Montserrat, Bustamante, et al. 2019. In the encoder, the layers progressively reduce in size, meaning each subsequent layer contains fewer neurons. Conversely, in the decoder, the layers expand, with each layer having a greater number of neurons in sequence. At the bottleneck of the architecture we find two feature maps: one trained to be the mean vector and another one the log-variance vector. These two feature maps, in conjunction with the VAE reparametrization trick, combine to yield the latent representation of the input $\boldsymbol{z}$. At each layer within the encoder, we apply batch normalization (Ioffe et al. 2015), an operation that enhances the flow of gradients throughout the network. Following each fully connected layer, a non-linearity is applied. We have experimented with a full set of different non-linearities, and the best results are achieved with *rectified linear units* (ReLUs) (Krizhevsky et al. 2017) and *Gaussian error linear units* (GELUs)

(Hendrycks et al. 2020), in that order. The final layer of the decoder utilizes the sigmoid activation function to produce the probability vector for SNP positions, denoted as $\boldsymbol{o}$. These resulting probabilities are clamped with a unit step function $\mathbb{1}_{1/2}(\cdot)$ applied elementwise to obtain the reconstruction of the input $\hat{\boldsymbol{x}}$. While our implementation allows the adjustment of the shape of the network, our current default settings use three dense layers of 512, 64 and 2 neurons, respectively, for the task of dimensionality reduction, and two dense layers of 512 and 64 neurons for all of the other tasks.

Supplemental Methods S6. Loss function

The loss function employed for training the VAE encompasses two primary objectives: the generative loss term and the latent loss term.

The generative loss objective strives for a high-quality reconstruction, aiming to make $\hat{\boldsymbol{x}}$ as close as possible to $\boldsymbol{x}$, so that $\hat{\boldsymbol{x}} \approx \boldsymbol{x}$. When we seek to maximize the probability of a reconstructed SNP position $x_i, 1 \leq i \leq d$ belonging to a known distribution with specific distribution parameters, our goal is to maximize the likelihood function for that particular distribution. In the **Results** section, we explain that each individual SNP can be effectively modeled with a Bernoulli distribution. Therefore, maximizing the likelihood for a Bernoulli distribution translates to minimizing the cross-entropy loss between $\boldsymbol{x}$ and $\boldsymbol{o}$.

Let $\boldsymbol{\theta}$ represent the parameters of the VAE model, denoted as $f_{\boldsymbol{\theta}}(\cdot)$, and let us denote the dataset $\mathcal{D}_x = \{\boldsymbol{x}_n | 1 \leq n \leq N\}$ as the set of d-dimensional samples. Assuming i.i.d. data samples from the distribution $p(\mathcal{D}_x)$, we can define a multinomial likelihood in the following form:

$$p(\mathcal{D}_x|\boldsymbol{\theta}) = \prod_{n=1}^{N} p(\boldsymbol{x}_n|\boldsymbol{\theta}) \tag{17}$$

The reconstruction loss function considers that the loss of each SNP is independent of each other. However, that does not imply the VAE models SNPs as independent. On the contrary, by using non-linearities, the VAE is able to model non-linear relationships between genetic positions. Therefore, we can approximate the likelihood function as follows:

$$
\begin{aligned}
p(\mathcal{D}_x|\boldsymbol{\theta}) &= \prod_{n=1}^{N}\prod_{i=1}^{d} f_{\boldsymbol{\theta}}(\boldsymbol{x}_n)_i^{x_{ni}} \left(1 - f_{\boldsymbol{\theta}}(\boldsymbol{x}_n)_i\right)^{(1-x_{ni})} \\
&= \prod_{n=1}^{N}\prod_{i=1}^{d} (o_{ni})^{x_{ni}} \left(1 - o_{ni}\right)^{(1-x_{ni})}
\end{aligned} \tag{18}
$$

Taking the logarithm on this expression, we obtain:

$$
\begin{aligned}
\log p(\mathcal{D}_x|\boldsymbol{\theta}) &= \sum_{n=1}^{N}\sum_{i=1}^{d} \Big[x_{ni} \log\left(o_{ni}\right) \\
&\quad + (1 - x_{ni}) \log\left(1 - o_{ni}\right) \Big] \\
&= -\sum_{n=1}^{N} \ell_{\mathrm{BCE}}(\boldsymbol{x}_n, \boldsymbol{o}_n)
\end{aligned} \tag{19}
$$

Thus, the generative loss for the VAE is computed as the *binary cross-entropy* (BCE) loss, ℓ_{BCE}, between each sample $\boldsymbol{x}_n$ and the VAE output $\boldsymbol{o}_n$ for that sample.

The latent loss encourages $\boldsymbol{z}$ to follow a standard Gaussian distribution, i.e., $\boldsymbol{z} \sim \mathcal{N}(\boldsymbol{0}, \boldsymbol{1})$. To compare the distribution of the latent vector with a zero-mean, unit-variance Gaussian distribution, we employ the *Kullback-Leibler* (KL) divergence. This results in $\mathcal{D}_{KL}\left(p(\boldsymbol{z}|\boldsymbol{x})||\mathcal{N}(\boldsymbol{0}, \boldsymbol{1})\right)$, where $\boldsymbol{z}|\boldsymbol{x} \sim \mathcal{N}(\boldsymbol{\mu}, \boldsymbol{\Sigma})$ is the encoder distribution defined by the two vectors at the bottleneck: the mean vector $\boldsymbol{\mu}$ and the logarithm of the variance vector diag($\boldsymbol{\Sigma}$), as $\boldsymbol{\Sigma}$ is assumed to be a diagonal covariance matrix. The latent loss acts as a regularizing term, which is weighted with variational β. The loss for a given input $\boldsymbol{x}$ and VAE-computed output $\boldsymbol{o}$ is given by:

$$
\begin{aligned}
\mathcal{J}_{VAE}(\boldsymbol{x}, \boldsymbol{o}) &= \ell_{\mathrm{BCE}}(\boldsymbol{x}, \boldsymbol{o}) + \beta \mathcal{D}_{KL}\Big(p(\boldsymbol{z}|\boldsymbol{x})||\mathcal{N}(\boldsymbol{0}, \boldsymbol{1})\Big) \\
&= \underbrace{\left[\sum_{i=1}^{d} x_i \log o_i + (1 - x_i)\log\left(1 - o_i\right)\right]}_{\text{Generative loss}} \\
&\quad - \beta \underbrace{\frac{1}{2}\left[\sum_{i=1}^{q} (\sigma_i + \mu_i^2 - \log \sigma_i)\right]}_{\text{Latent loss}}
\end{aligned} \tag{20}
$$

where d denotes the dimensionality of the observed data, $\boldsymbol{x}$, while q represents the dimensionality of the latent representation, $\boldsymbol{z}$. In our experiments, the landscape of $\mathcal{L}_{VAE}$ is traversed by the quasi-hyperbolic momentum variant of Adam (QHAdam) optimizer (Ma et al. 2019).

Supplemental Methods S7. Clustering metrics

To quantitatively compare the quality of population groups in both, PCA and VAE spaces, we employ three clustering metrics: the *pseudo F statistic* (also known as Calinski-Harabasz index) (Caliński et al. 1974), the *Davies-Bouldin index* (DBI) (Davies et al. 1979), and the *silhouette coefficient* (SC) (Kaufman et al. 2009). Pseudo F statistic measures the ratio between the sum of between- and within-cluster dispersion (BSS and WSS, respectively):

$$\begin{aligned} \mathrm{CH}(|\mathcal{Y}|) &= \frac{\mathrm{BSS}(|\mathcal{Y}|)}{\mathrm{WSS}(|\mathcal{Y}|)} \cdot \alpha(|\mathcal{Y}|) \\ &= \frac{\sum_{k=1}^{|\mathcal{Y}|} \sum_{n=1}^{N} \mathbb{1}_{nk} ||\boldsymbol{\mu}_k - \bar{\boldsymbol{x}}||_2^2}{\sum_{k=1}^{|\mathcal{Y}|} \sum_{n=1}^{N} \mathbb{1}_{nk} ||\boldsymbol{\mu}_k - \boldsymbol{x}_n||_2^2} \cdot \frac{N - |\mathcal{Y}|}{|\mathcal{Y}| - 1} \end{aligned} \tag{21}$$

where the number of clusters coincides with the number of populations, denoted as $|\mathcal{Y}|$; N represents the number of samples in the dataset; $\mathbb{1}_{nk}$ serves as an indicator function, determining whether sample $\boldsymbol{x}_n$, $1 \leq n \leq N$, belongs to ancestry label k, with $1 \leq k \leq |\mathcal{Y}|$; $\bar{\boldsymbol{x}}$ represents the mean of the samples, and $\boldsymbol{\mu}_k$ denotes the centroid of the k-th ancestry. A higher score indicates that the clusters are more compact and well-separated. Conversely, DBI inversely relates to cluster separation; a smaller DBI value suggests better separation between clusters as it computes the maximal ratio between intra-cluster variance and inter-cluster distance (Davies et al. 1979):

$$\begin{aligned} \mathrm{DBI}(|\mathcal{Y}|) = \frac{1}{Y} \sum_{k=1}^{|\mathcal{Y}|} \max_{k \neq j} \Bigg[& \frac{\sum_{n=1}^{N} \mathbb{1}_{nk} ||\boldsymbol{\mu}_k - \boldsymbol{x}_n||_2^2}{||\boldsymbol{\mu}_k - \boldsymbol{\mu}_j||_2^2} \\ & + \frac{\sum_{n=1}^{N} \mathbb{1}_{nj} ||\boldsymbol{\mu}_j - \boldsymbol{x}_n||_2^2}{||\boldsymbol{\mu}_k - \boldsymbol{\mu}_j||_2^2} \Bigg] \end{aligned} \tag{22}$$

Lastly, SC relates the intra-cluster distance of samples and the nearest-cluster distance. This coefficient is bounded $\mathrm{SC}(|\mathcal{Y}|) \in [-1, 1]$. A value of $\in [0.71, 1]$ is an indicator of a strong structure in the data. Values below < 0.25 indicate that no substantial structure has been found (Kaufman et al. 2009).

Supplemental Methods S8. VQ-VAE training configuration and run times

All VQ-VAE models were trained on NVIDIA GeForce RTX 2080 Ti GPUs (11 GB). A single GPU completed training in roughly 16 minutes for the 10,000-SNP model and 1

hour 8 minutes for the 50,000-SNP model. Training the 80,000-SNP model on one GPU required about 2 hours. For the full Chromosome 22 model (317,000 SNPs), we parallelised the run across four RTX 2080 Ti cards, reducing wall-clock time to approximately 2 hours.

Supplemental Results S1. Compression factors of PCA versus ancestry-conditioned VAE

In this setup, the key difference is the inclusion of an additional explanatory variable, denoted as y, which encodes the ancestry or breed of the subject. In practical terms, this implies that before compressing a set of SNP arrays, we explicitly condition the encoder based on ancestry or breed. Similarly, during the expansion step, we condition the decoder using that same label. Following our experiments, we opted to continue with the conventional VAE. This decision was based on the slightly worse compression performance observed with C-VAE and its inability to effectively compress the African (AFR) population. A comparison between VAE and C-VAE is provided in Supplemental Table 3.

Supplemental Table S3. Compression factors of VAE versus C-VAE. The compression factors are computed as $\frac{\ell(\boldsymbol{x})}{\ell(\boldsymbol{z})+\ell(\mathcal{A}(\boldsymbol{r}))}$ using test data. A compression ratio of 1 corresponds to the identity, values < 1 and > 1 correspond to compression and expansion, respectively. Successful compression is marked in *bold*.$|\boldsymbol{z}|$ is the number of latent factors and α stands for *learning rate*.

**Models**			**Populations**						
**Type**	$\lvert\boldsymbol{z}\rvert$	$\boldsymbol{\alpha}$	**European (EUR)**	**East Asian (EAS)**	**Native American (AMR)**	**South Asian (SAS)**	**African (AFR)**	**Oceanian (OCE)**	**West Asian (WAS)**
VAE	2	10^{-4}	×0.68	×0.64	×0.76	×0.62	×0.53	×0.50	×0.67
C-VAE			×0.57	×0.54	×0.58	×0.54	×0.43	×0.46	×0.55
VAE	4	10^{-4}	×0.77	×0.69	×0.87	×0.68	×0.56	×0.58	×0.71
C-VAE			×0.63	×0.58	×0.63	×0.58	×0.42	×0.51	×0.58
VAE	8	10^{-4}	×1.00	×0.93	**×1.17**	×0.88	×0.63	×0.68	×0.89
C-VAE			×0.78	×0.71	×0.73	×0.69	×0.45	×0.56	×0.70
VAE	16	10^{-4}	**×1.59**	**×1.39**	**×1.73**	**×1.32**	×0.84	**×1.01**	**×1.37**
C-VAE			**×1.02**	×0.77	×0.90	×0.88	×0.50	×0.65	×0.90
**VAE**	**32**	$\boldsymbol{10^{-4}}$	**×2.00**	**×1.75**	**×2.33**	**×1.69**	**×1.03**	**×1.27**	**×1.72**
C-VAE			**×1.32**	**×1.10**	**×1.23**	**×1.12**	×0.59	×0.85	**×1.14**
**VAE**	**64**	$\boldsymbol{10^{-4}}$	**×2.04**	**×1.82**	**×2.27**	**×1.75**	**×1.16**	**×1.47**	**×1.82**
C-VAE			**×1.32**	**×1.20**	**×1.30**	**×1.18**	×0.63	×0.88	**×1.18**
**VAE**	**128**	$\boldsymbol{10^{-4}}$	**×1.54**	**×1.45**	**×1.61**	**×1.41**	**×1.06**	**×1.25**	**×1.43**
C-VAE			**×1.27**	**×1.20**	**×1.28**	**×1.19**	×0.77	**×1.01**	**×1.20**
VAE	256	10^{-4}	×0.97	×0.93	×1.00	×0.93	×0.79	×0.86	×0.93
C-VAE			×0.90	×0.86	×0.91	×0.86	×0.68	×0.78	×0.87
VAE	512	10^{-4}	×0.54	×0.53	×0.55	×0.53	×0.48	×0.50	×0.53
C-VAE			×0.52	×0.50	×0.52	×0.50	×0.44	×0.48	×0.51

Supplemental Results S2. Synthetic genotypes enable accurate downstream ancestry classification

To quantify the downstream utility of the genotypes generated by our VAE, we performed a multiclass ancestry-classification benchmark in which synthetic data served as training material and real founder genotypes provided an independent test set. Genotypes were first projected onto their first 50 principal components using a PCA transformation fitted to founder genotypes. We then train five standard classifiers (logistic regression, random forest, Support Vector Machines (SVM), gradient boosting, and Naive Bayes) on the PCA-transformed Wright-Fisher (WF) and VAE data (each with 19,600 samples), and evaluate their performance on PCA-transformed real founder genotypes. The results, shown in Supplemental Figure 1, demonstrate that classifiers trained on VAE-simulated data achieve accuracy levels that are consistently close to those trained on WF-augmented genotypes across a diverse set of model classes. This supplemental result suggests that synthetic genotypes generated by our VAE approach capture meaningful population structure and support downstream ancestry inference.

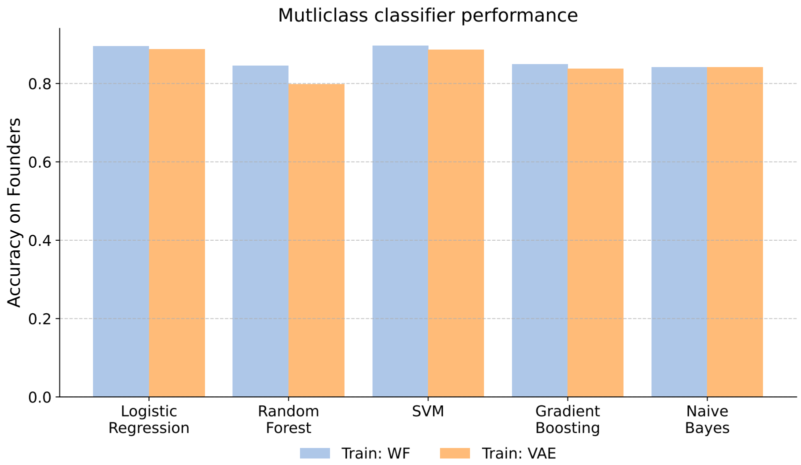

Supplemental Figure S1. Multiclass classifier comparison. Classification accuracy on the first 50 principal components of real founder genotypes using models trained on the first 50 principal components of a genotype dataset augmented by standard Wright-Fisher (WF) simulation, and on the first 50 principal components of a VAE-generated genotype dataset (both with 19,600 samples). While classifiers trained on Wright-Fisher data serve as a baseline, those trained on VAE-generated data achieve comparable performance across a range of model classes, indicating the utility and structural realism of the VAE simulations for downstream ancestry inference tasks.

Supplemental Results S3. Comparison of GMMN and VAE for genotype simulation

GMMN are architecturally distinct from VAEs, as well as their objective criterion—GMMNs rely on matching the statistical moments of real data in the artificial samples via Maximum Mean Discrepancy (MMD) minimization. Because training does not require a discriminator (as in GANs) or an encoder (as in VAEs), GMMNs are in principle conceptually simpler and Perera et al. 2022 argue that this simplicity makes them easier to train. However, in practice, GMMNs with random features are computationally restrictive for high-dimensional genotype data. Their loss requires computing random feature embeddings for each population and retaining large computational graphs for compute amortization. This leads to high GPU memory usage and limited scalability. Consistent with the original GMMN paper, we therefore restricted training to 5,000 SNPs and used 10,000 training samples. This was sufficient for comparison, but substantially smaller than the genome-wide scale of our VAE experiments.

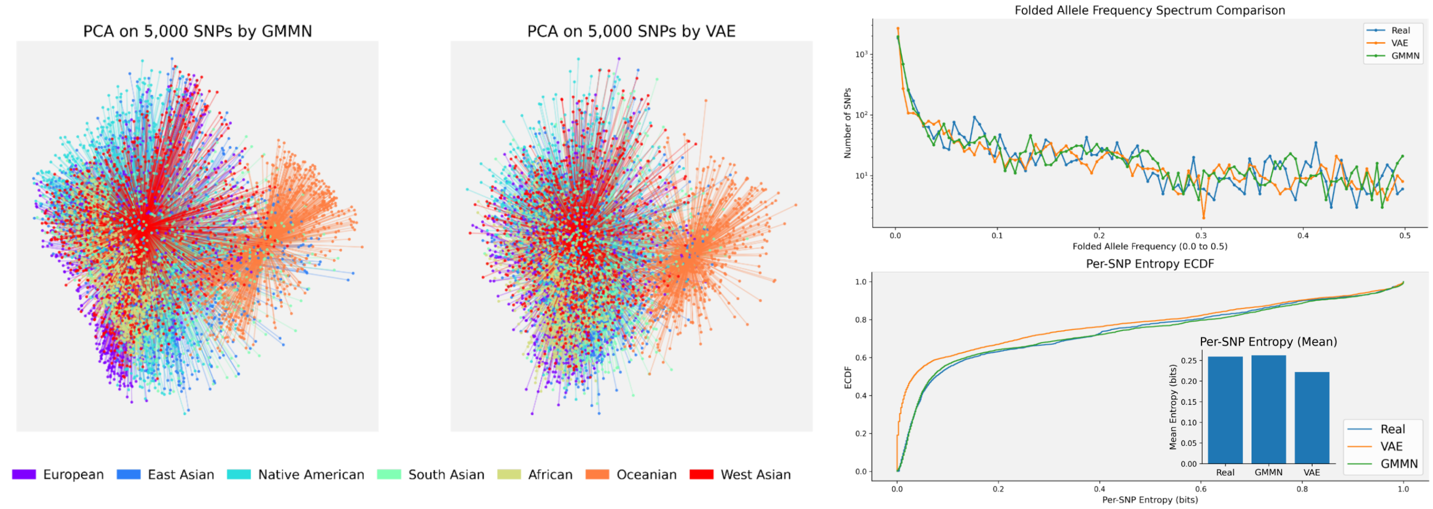

Supplemental Figure S2. Comparison of real genotype data with simulated data generated by GMMN and VAE models. *(Left Panels)* PCA projections of 5,000 SNPs (human Chromosome 22) generated by GMMN and VAE, colored by population labels. Principal components were fitted on real 5,000 SNPs founder samples. *(Top-right)* Folded allele frequency spectrum comparing real and simulations by GMMN and VAE. *(Bottom-right)* empirical CDF of per-SNP entropy distributions, illustrating how the cumulative distribution of uncertainty in GMMN and VAE simulations tracks the real data.

We trained both GMMN and conditional VAE on the same subset. Results are shown in Supplemental Figure S2. Qualitatively, both methods recover broad ancestry

structure; cluster locations are similar, with C-VAE clusters appearing smoother, consistent with a Gaussian latent prior. The reduced SNP set naturally weakens separation compared to genome-wide panels but still reveals clear population stratification. In terms of folded AFS, both models recover the overall allele frequency spectrum of the real founders, including the characteristic excess at low minor-allele frequencies. We also computed the empirical CDF of per-SNP Bernoulli entropy and it exposes subtle differences in the low-entropy tail. The C-VAE curve rises earlier than real, indicating a slightly higher fraction of near-fixed sites (conservative bias; mild underestimation of diversity at some loci). The GMMN curve is slightly below real at the left tail and catches up in the mid-range, indicating fewer near-fixed sites and a mild shift toward moderate entropies (slight overdispersion at some loci, injecting some extra noise). Both models align well with real data from mid to high entropy, suggesting overall diversity levels are well captured. In short, GMMN and C-VAE reproduce the global entropy profile while differing in how aggressively they model low-entropy SNPs.

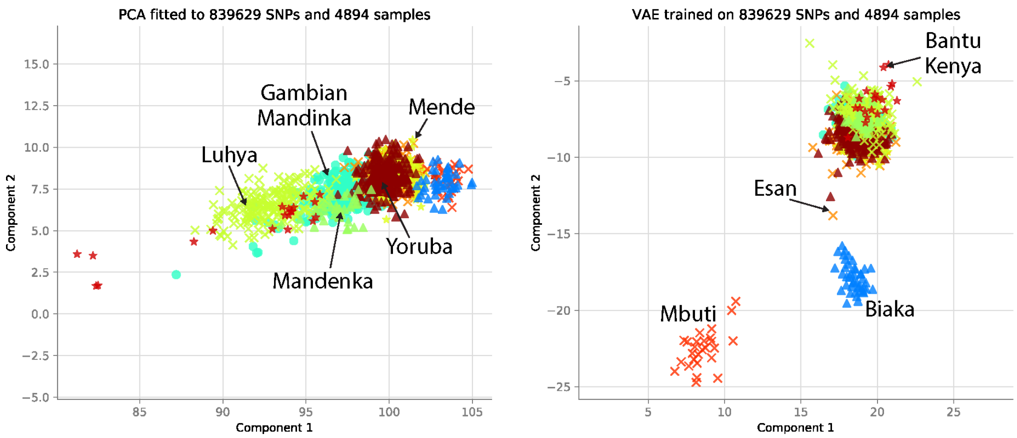

Supplemental Figure S3. Comparison of PCA and VAE projections of African subpopulations. VAE trained genome-wide using 839,629 SNPs and no admixture between subpopulations. The VAE clusters highlight clear separation of the Mbuti and Biaka groups from the central African cluster, indicating that the model effectively captures fine-grained population structure beyond what is observable through PCA alone.

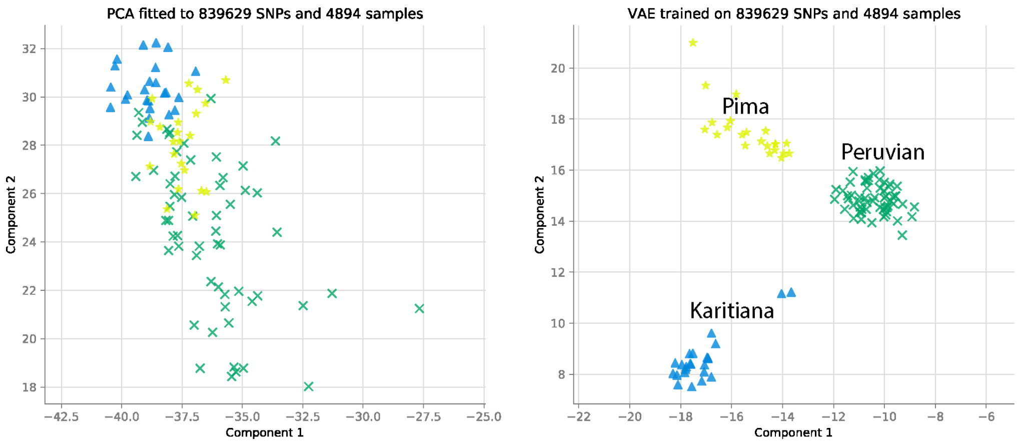

Supplemental Figure S4. Comparison of PCA and VAE projections of Native American-like subpopulations. Notably, the VAE produces more compact and distinct clusters for each subpopulation, highlighting its capacity to capture subtle genetic differences between subpopulations: Pima (yellow stars), Karitiana (blue triangles), and Peruvian (green crosses).

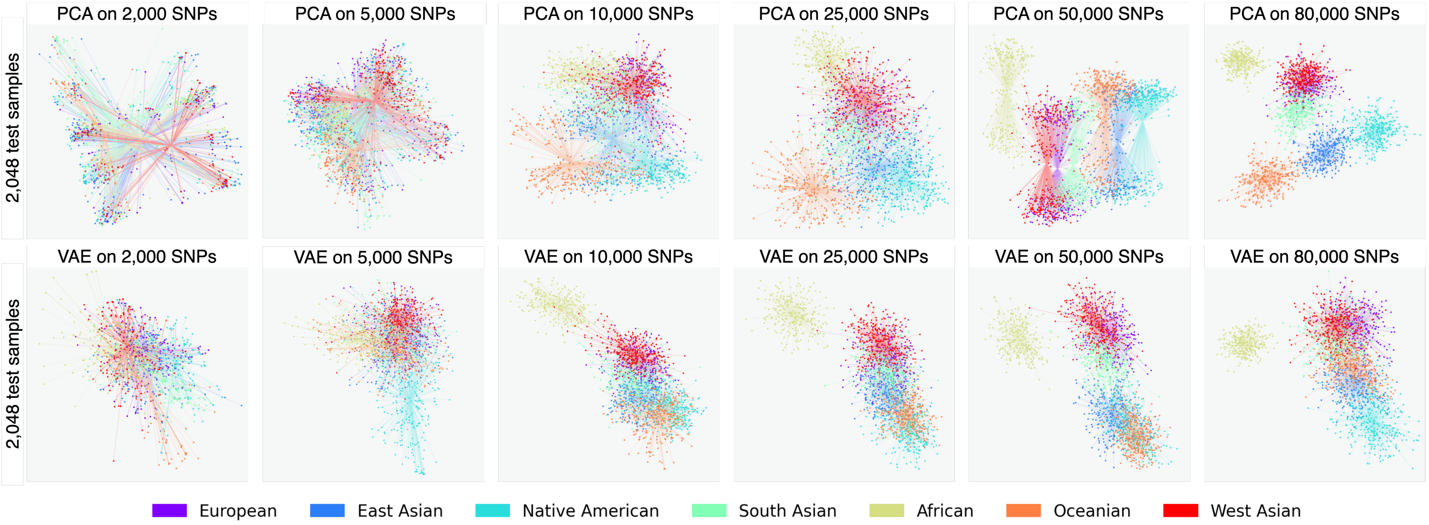

Supplemental Figure S5. Comparison of PCA and VAE with a bottleneck in $\mathbb{R}^{64}$ applied to varying lengths of sequential SNP sequences. VAE models trained genome-wide using different subsets of SNPs (2,000; 5,000; 10,000; 25,000; 50,000; 80,000) with admixture between subpopulations.

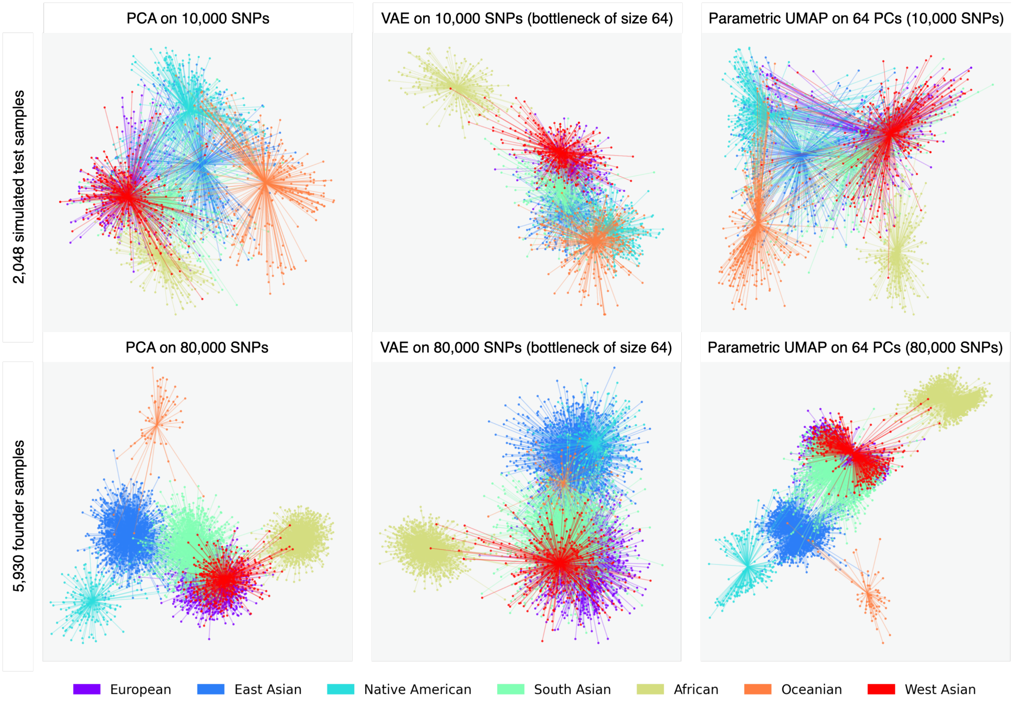

Supplemental Figure S6. Comparison of PCA, VAE, and UMAP projections. (a) First row: PCA, VAE, UMAP projections of 10,000 SNP positions of human Chromosome 22 using simulated samples; (b) Second row: PCA, VAE, UMAP projections of 80,000 SNP positions of human Chromosome 22 using (real) founder samples.

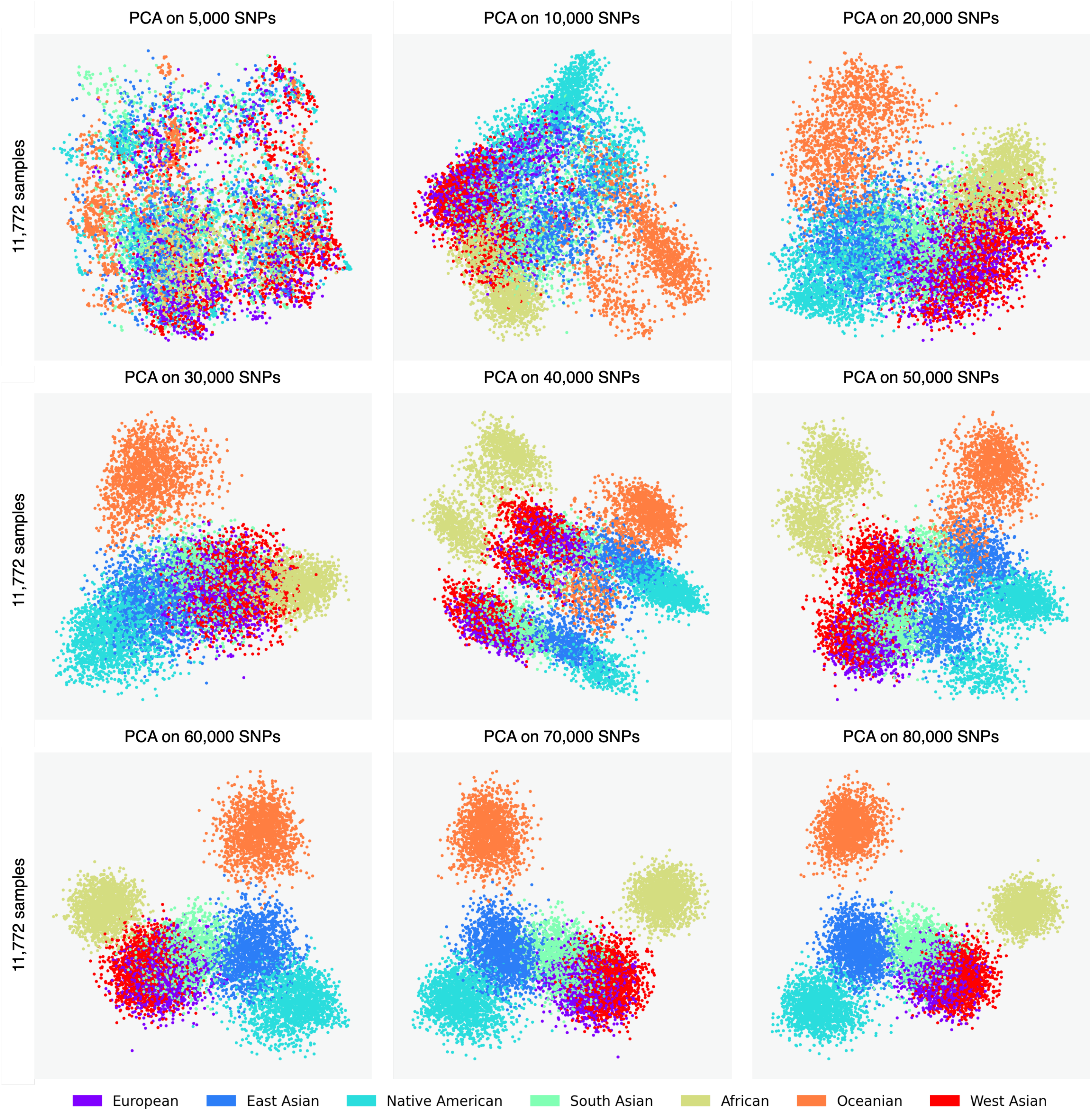

Supplemental Figure S7. Visual effect of LD on PCA projections. We use subsets of sequential SNPs from human Chromosome 22 of sizes 5,000; 10,000; 20,000; 30,000; 40,000; 50,000; 60,000; 70,000, and 80,000 SNPs. The *polarization effect* disappears when we use an effectively large subsample of sequential SNPs.

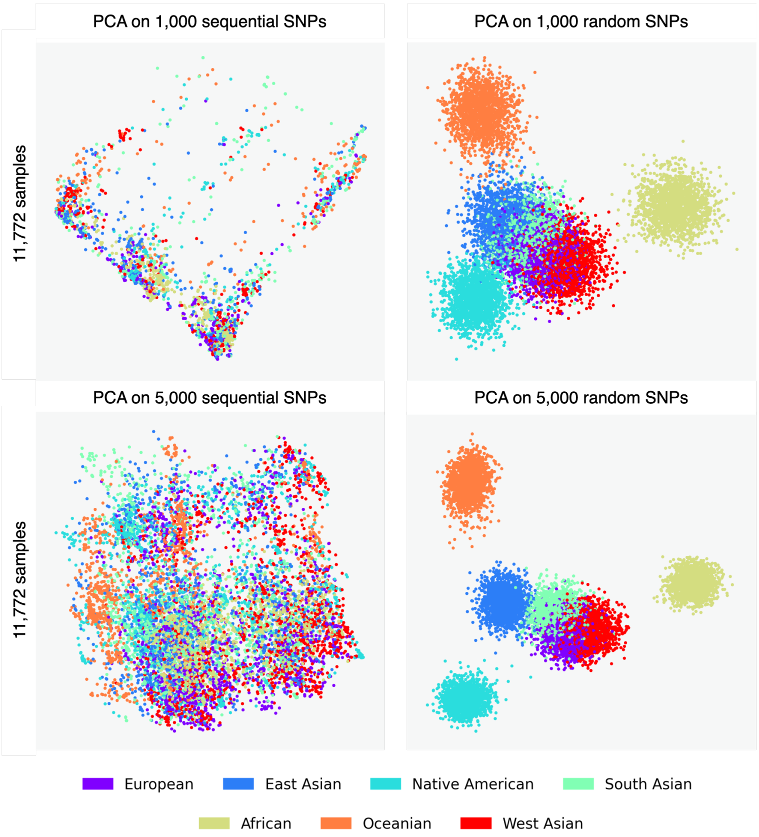

Supplemental Figure S8. PCA on a subset of random and sequential SNPs. The visual difference is due to LD structure.

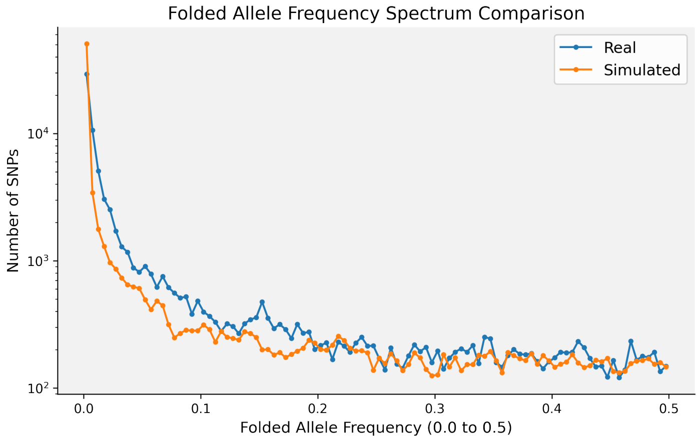

Supplemental Figure S9. Folded allele frequency spectrum, showing the proportion of SNPs at each minor allele frequency for both, real and simulated data with a C-VAE model trained genome-wide on 80,000 SNPs on human Chromosome 22. The y-axis is shown on a logarithmic scale. The result suggests that our simulated data reflects patterns found in real data.

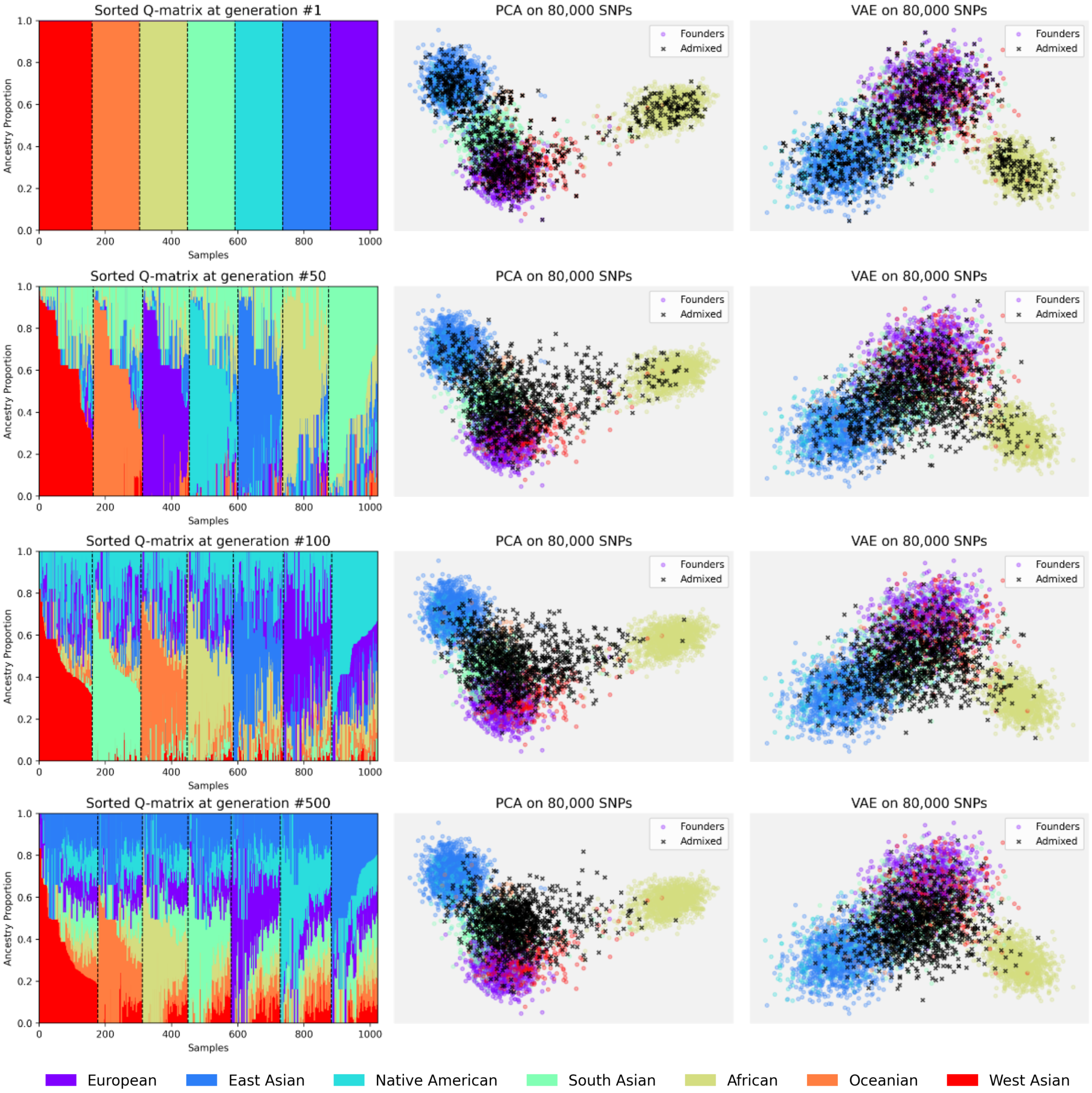

Supplemental Figure S10. Comparison of how PCA and VAE represent admixed individuals across generations of admixture. *(Left)* Color-coded ancestry estimates of simulated admixed samples; *(Center and Right)* Simulated admixed samples are represented in black over the color-coded latent representation of single-ancestry individuals (see legend). *(Center)* the same samples embedded via PCA, and *(Right)* their embedding using a VAE.
